# Changes in Gut Microbiota Prior to Influenza A Virus Infection Do Not Affect Immune Responses in Pups or Juvenile Mice

**DOI:** 10.3389/fcimb.2018.00319

**Published:** 2018-09-12

**Authors:** Eva Fuglsang, Angela Pizzolla, Lukasz Krych, Dennis S. Nielsen, Andrew G. Brooks, Hanne Frøkiær, Patrick C. Reading

**Affiliations:** ^1^Department of Veterinary and Animal Sciences, University of Copenhagen, Frederiksberg, Denmark; ^2^Department of Microbiology and Immunology, The Peter Doherty Institute for Infection and Immunity, The University of Melbourne, Melbourne, VIC, Australia; ^3^Department of Food Science, University of Copenhagen, Frederiksberg, Denmark; ^4^The Peter Doherty Institute for Infection and Immunity, WHO Collaborating Centre for Reference and Research on Influenza, Melbourne, VIC, Australia

**Keywords:** influenza, antibiotics, gut microbiota, adaptive immunity, juvenile mice, mouse pups

## Abstract

Previous studies demonstrated that oral antibiotic (ABX) treatment prior to and during influenza A virus (IAV) infection of adult mice profoundly altered gut microbiota (GM) and was associated with increased susceptibility and impaired immunity to IAV. We examined the impact of ABX during critical times relevant to the establishment of GM in early life (using perinatal treatment of neonates and direct treatment of juvenile mice) and asked whether cessation of ABX treatment in early life had lasting effects on GM composition and anti-IAV immunity. ABX treatment significantly changed GM composition in juvenile mice and in ABX-treated dams. However, if ABX treatment ceased at the time of infection, neither neonates nor juvenile mice showed enhanced susceptibility to IAV, nor were major differences detected in cellular and humoral adaptive antiviral immunity. Thus, while ABX treatment alters GM diversity in early life, cessation and subsequent re-colonization correlates with effective immunity against IAV.

## Introduction

Seasonal influenza virus infections affect 10-20% of the world's population every year (Peasah et al., 2013), of which young children and elderly people are especially vulnerable to influenza-associated disease and death [reviewed in (Thompson et al., 2003; Moghadami, 2017)]. Although influenza is generally an acute self-limiting infection that can be treated with appropriate antiviral drugs, surveys and studies in the USA (Nyquist et al., 1998; Brown et al., 2003; Ciesla et al., 2004) and in Europe (Ochoa et al., 2000; Mazzaglia et al., 2003; Tsolia et al., 2006; Ploin et al., 2007) have shown that antibiotics (ABX) are often used to treat a number of respiratory virus infections, even when there is no evidence of bacterial co-infections (Ochoa et al., 2000; Mazzaglia et al., 2003; Ciesla et al., 2004; Tsolia et al., 2006; Ploin et al., 2007). This is of major concern, particularly given recent evidence of increased microbial resistance to ABX worldwide (reviewed in Ventola, 2015). Further, research in mouse models highlights that the impact of ABX treatment on the composition of gut microbiota (GM) correlates with enhanced susceptibility to influenza virus-associated morbidity and mortality (Ichinohe et al., 2011; Abt et al., 2012). Although the underlining mechanisms are not fully understood, studies in adult mice treated with ABX demonstrated that increased susceptibility correlated with impaired type I interferon (IFN) signaling pathways in airway macrophages (Abt et al., 2012). Furthermore, influenza virus-specific cytotoxic T cells did not differentiate or proliferate due to impaired inflammasome-dependent migration of dendritic cells (DCs) from the lung to mediastinal lymph nodes (medLN) (Ichinohe et al., 2011). Thus, studies in the mouse model highlight the importance of minimizing the use of ABX in human patients presenting with influenza infections, particularly when bacterial coinfection has not been confirmed.

Studies in mice have characterized the colonization of the gut following birth, investigating the influence of genetic background (Friswell et al., 2010; Hufeldt et al., 2010a; Buhnik-Rosenblau et al., 2011), *in utero* maternal colonization with gut microbes (Jiménez et al., 2008) and diet (Zhang et al., 2010) in shaping the GM composition (Hufeldt et al., 2010b; Deshmukh et al., 2014). In mice, the GM composition is thought to stabilize shortly after weaning at 3 weeks of age (Hirayama et al., 1995). It has been established that ABX-induced changes in GM diversity during adulthood are transient and tend to recover, presumably due to the dynamic nature of the established GM (Antonopoulos et al., 2009; Croswell et al., 2009). However, ABX treatment of mice during weaning or at the time of colonization, or when mice are held in germ-free conditions, has been associated with long-term effects on GM composition and lymphocyte development (Hansen et al., 2012, 2013). Thus, post-partum colonization with maternal-derived bacteria up until and during weaning plays an essential role in development of the immune system. Moreover, disruptions to this process by ABX treatment early in life can impair aspects of immunity, including development of effector T cell responses (Hill and Artis, 2010; Reading and Kasper, 2011; Chung et al., 2012).

Studies investigating the impact of ABX-induced changes on GM composition and susceptibility to influenza A virus (IAV) infection in mice have focused on the impact of ABX treatment during infection of adult animals, which resulted in exacerbated disease (Ichinohe et al., 2011; Abt et al., 2012; Gonzalez-Perez et al., 2016; Gonzalez-Perez and Lamousé-Smith, 2017). Our studies have therefore focused on investigating how ABX treatment prior to, but not during, IAV infection impacts the GM composition, as well as the development of disease and immunity following subsequent IAV infection. To broaden the scope of our study, we have examined the effects of ABX treatment at critical time points relevant to the establishment of GM composition in young animals. First, we assessed the impact of direct ABX treatment in weaning juvenile mice, given this time is reported to be associated with stabilization of the GM (Hirayama et al., 1995). Second, we examined effects of perinatal ABX treatment of pregnant dams on GM composition and antiviral immunity of their pups, given that the GM composition of the dam is known to be a major factor in determining colonization of pups (Fåk et al., 2008; Gonzalez-Perez et al., 2016).

## Materials and methods

### Ethics approval statement

Experiments using mice were conducted with approval from the University of Melbourne Biochemistry and Molecular Biology, Dental Science, Medicine, Microbiology and Immunology, and Surgery Animal Ethics Committee (project 1413227.3), in accordance with the National Health and Medical Research Council (NHMRC) Australian code of practice for the care and use of animals for scientific purposes.

Experiments using 10-day embryonated chicken eggs were conducted with approval from the University of Melbourne Biochemistry and Molecular Biology, Dental Science, Medicine, Microbiology and Immunology, and Surgery Animal Ethics Committee (project 1714213), in accordance with the National Health and Medical Research Council (NHMRC) Australian code of practice for the care and use of animals for scientific purposes. Eggs were obtained from Hy-Line Australia (Bagshot, Victoria, Australia).

### Virus

IAV strain X31 is a high-yielding reassortant of A/PR/8/34 (PR8; H1N1) and A/Aichi/2/1968 (H3N2), which expresses the H3N2 HA and NA surface glycoproteins. X31 was grown in 10-day embryonated hen's eggs by standard procedures (Job et al., 2014) and stored in aliquots at −80°C prior to use. Titres of infectious virus were determined by standard plaque assay on Madin-Darby canine kidney (MDCK) cells (Job et al., 2014), and are expressed as plaque-forming units (PFU)/ml.

### Antibiotic treatment and IAV infection of mice

C57BL/6 male and female mice were bred, housed and mated in specific pathogen-free facilities at the Peter Doherty Institute for Infection and Immunity, Department of Microbiology and Immunology, University of Melbourne, Australia. Pregnant dams were single-housed after confirmed pregnancy (2 weeks pregnant) and the pups were weaned at 3 weeks of age.

#### Juveniles

Following weaning at 3 weeks of age, juvenile mice were randomly assigned to receive either (i) normal drinking water, or (ii) drinking water supplemented with ampicillin (0.5 mg/ml), gentamicin sulfate (0.5 mg/ml), and metronidazole (0.5 mg/ml) (all from Sigma Aldrich, collectively called ABX). Mice were treated for 3 weeks (ABX1) or 2 weeks followed by a week on normal drinking water (ABX2) and ABX water was changed every third day. A control group received normal drinking water throughout this time (water). After 2 weeks of ABX treatment for ABX1/ABX2 groups (i.e., 5 weeks of age), all mice were anesthetized using isoflurane and infected via the intranasal (i.n.) route with 100 PFU of X31 in 50 μl phosphate-buffered saline (PBS). Control mice were mock-infected with 50 μl of PBS. ABX and water groups were randomly assigned to be IAV- or mock infected. Mice were weighed daily from day 1 to 10 post-infection (p.i.) and assessed for signs of clinical disease. Animals that had lost >15% of their original body weight and/or displayed evidence of pneumonia were euthanized.

#### Pups

Following confirmed pregnancy (2 weeks pregnant), pregnant dams were single housed and randomly assigned to receive either (i) normal drinking water, or (ii) drinking water supplemented with ABX as described above. Dams were treated for 2 weeks (1 week pre-partum to 1 week post-partum) and ABX water was changed every third day. After 2 weeks with or without ABX treatment, all dams received normal drinking water. For i.n. infection with X31, 1 week old pups received 20 PFU in 3 μl PBS and 2 weeks old pups received 100 PFU in 10 μl PBS without anesthesia whereas 5 weeks old pups were anesthetized using isoflurane and received 100 PFU in 50 μl PBS. Age-matched mice of 1, 2, or 5 weeks of age were mock-infected with 3, 10 or 50 μl of PBS, respectively. ABX and water pup groups were randomly assigned to be IAV- or mock infected. Pups were weighed daily from day 1 to 10 p.i. and assessed for signs of clinical disease. In pups infected with X31, one or more of the following criteria resulted in euthanasia; absence of milk spot (> 12 h, with dehydration), isolation from littermates, piloerection, head tilt, labored breathing or no weight gain compared to littermates (after 24 h with gel pack).

### Titres of virus in tissues from virus-infected mice

Nasal tissues and lungs were collected from IAV-infected mice on day 7 p.i. unless otherwise stated. Tissues were homogenized in 1 ml of PBS, clarified by centrifugation and stored at −80°C. Titres of infectious virus were determined by standard plaque assay on MDCK cells and expressed as PFU/organ.

### Fecal sample collection and microbial DNA extraction

#### Juvenile

Fecal samples were collected on the day of infection (day 0 p.i. when mice were 5 weeks of age) and immediately prior to when they were killed for analysis at day 7 p.i. (when mice were 6 weeks of age). Samples were stored at −80°C prior to DNA extraction.

#### Pups

Fecal samples were collected from pregnant dams on the day that ABX treatment ceased and were stored at −80°C prior to DNA extraction.

Fecal samples were homogenized and DNA was extracted using a MoBio Power Soil Kit (MoBio Laboratories, QIAGEN). Samples were stored at −80°C prior to further analysis as described below.

### Isolation of cells from spleen, lung tissue and mediastinal lymph nodes

Mice were euthanized by carbon dioxide inhalation (100% CO_2_ at slow fill rate of 20% of the chamber volume/minute) and lungs were perfused with PBS prior to collection. The spleen and medLNs were also collected from animals. Lungs were minced and incubated with Collagenase 3 (Scimar CLS-3, Worthington) and DNase I (Roche) in serum-free RPMI at 37°C for 60 min, then passed through a cell strainer (FALCON). Single cell suspensions were also prepared from spleen and medLNs by mechanical disruption through a cell strainer (FALCON). Cell suspensions from lungs, spleen and medLNs were treated with Red Blood Cell Lysing buffer (Sigma Aldrich) to lyse erythrocytes and then cell number and viability were determined using a hemocytometer and tryphan blue exclusion. Cell suspensions were then centrifuged and cells resuspended in FACS buffer [PBS, 2 mM EDTA, 2% FCS (Gibco)] to assess expression of cell-surface markers or in c-RPMI [RPMI 1640 (Gibco), 1 mM sodium pyruvate (Gibco), 2 mM L-glutamine (Gibco), 100 U/mL penicillin, 100 μg/mL streptomycin (Media Unit at Peter Doherty Institute, Melbourne), 5% FCS] for peptide stimulation followed by intracellular cytokine staining, as described below.

### Flow cytometry for cell surface markers and intracellular staining for cytokine expression

Single-cell suspensions prepared from lungs, spleen and medLNs were stained in FACS buffer with fluorescent-labeled antibodies to surface antigens and fixed in 1% paraformaldehyde. The following fluorochrome-conjugated antibodies were used: CD45.2-FITC (clone: 104), CD4-AF700 (clone: RM4-5), IFNγ-FITC (clone XMG1.2), TNFα-PE (MP6-XT22), and IL-2-APC (JES6-5H4) (all from BD Pharmingen); CD3ε-PercPCy5.5 (clone: 145-2C11), CD8α-PE-cy7 (clone: 53-6.7), Fixable viability dye-APC-Cy7 (eBioscience); NK1.1-BV711 (clone: PK136), CD44-BV650 (clone: IM7), CD62L-BV605 (clone: MEL-14) (BioLegend). To detect influenza-specific CD8 T cells, cells were stained with MHCI-peptide tetramers H2-D^b^NP_366−374_ conjugated with PE or H2-D^b^PA_224−233_ conjugated with APC for 1 hr at room temperature prior to staining with extracellular antibodies. Samples were collected with LSRFortessa flow cytometer (BD Biosciences) and analyzed by FlowJo v 10.1 (Treestar).

To assess intracellular expression of cytokines, cell suspensions incubated with or without 1 μM PA_224−233_ or NP_336−374_ peptide (Mimotopes) in presence of 25 U/ml recombinant IL-2 (Roche, Lifescience) and GolgiPlug (Brefeldin A, BD Biosciences Pharmingen) at 37°C for 5–6 h. After incubation, cells were washed, stained with fluorescent-labeled antibodies [CD3ε-PercPCy5.5 (clone: 145-2C11), CD8α-PE-cy7 (clone: 53-6.7), Fixable viability dye-APC-Cy7 (eBioscience)] and fixed in 1% paraformaldehyde. Cells were then permeabilized in FACS buffer containing 0.1% saponin (Sigma-Aldrich) and stained with antibodies [IFNγ-FITC (clone XMG1.2), TNFα-PE (MP6-XT22), and IL-2-APC (JES6-5H4), all from BD Pharmingen] to allow for detection of intracellular cytokine expression. Samples were collected and analyzed as for cell-surface staining. Gating strategies for surface and intracellular stains are shown in Figure S2.

### Serology assays - enzyme-linked immunosorbent assay (ELISA) to detect anti-IAV antibodies and hemagglutination inhibition (HI) assay

In some experiments, mice were killed at day 28 p.i. and blood was collected immediately via cardiac bleed. Samples were stored at 4°C overnight (O/N), then centrifuged and serum was collected and stored at −20°C prior to use in serology assays.

For ELISA, 96-well ELISA plates (Nunc Maxisorp) were coated O/N at 4°C with 0.5 μg/ml purified X31 in PBS containing 0.1% NaN_3_ (PBSN_3_) in a volume of 50 μl/well. Plates were then blocked O/N at room temperature with 100 μl/well containing 10 mg/ml bovine serum albumin (BSA) in PBSN_3_ then washed in PBS 0.05% (vol/vol) Tween 20 PBS (PBST). Serial ½ log dilutions of mouse sera were prepared in PBST containing 5 mg/ml BSA (BSA_5_PBST), and added to 96-well plates at 50 μl/well and incubated O/N at room temperature. After washing, all wells received 50 μl/well of rabbit anti-mouse IgG/IgM-HRP (Dako) in BSA_5_PBST and incubated for 2 hr at room temperature. Plates were then washed before addition of TMB substrate (BD OptEIA, BD Biosciences). The color reaction was stopped by addition of 50 μl/well of 1 M sulfuric acid before optical density was determined on a Multiskan Ascent microplate reader (Thermo Fisher Scientific) at 450 and 570 nm.

Hemagglutination titrations and HI assay were performed by standard procedures in 96-well plates with 1% (v/v) of turkey red blood cells. Briefly, hemagglutination titrations were used to adjust stocks of X31 virus to contain 4 hemagglutination units (HAU)/25 μl. HI assays were then performed using serum samples that had been heat inactivated at 56°C for 30 min. Two-fold dilutions of serum were performed in 25 μl PBS prior to addition of 4 HAU in 25 μl. After the addition of 25 μl of 1% turkey red blood cells, HI titres were determined and expressed as the highest serum dilution required to fully inhibiting the hemagglutination activity of 4 HAU of X31.

### Gut microbiota composition determination by 16S rRNA gene amplicon sequencing

Fecal microbiota composition of 56 C57BL/6 juvenile mice (water, *n* = 28; ABX1, *n* = 23; ABX2: *n* = 5) and 36 C57BL/6 dams (water, *n* = 18; ABX, *n* = 18) was determined using tag-encoded 16S rRNA gene (V3 region) NextSeq-based (Illumina, CA, USA) high throughput sequencing. Sequencing library preparation steps were conducted as previously described (Williams et al., 2017).

#### Data analysis

The raw dataset containing pair-ended reads with corresponding quality scores were merged and trimmed using settings as previously mentioned (Williams et al., 2017). Quantitative Insight Into Microbial Ecology (QIIME) open source software package (Caporaso et al., 2010) (1.7.0, 1.8.0, 1.9.0) was used for subsequent analysis steps. Purging the dataset from chimeric reads and constructing de novo Operational Taxonomic Units (OTU) was conducted using the UPARSE pipeline (Edgar, 2013). The green genes (13.8) 16S rRNA gene collection was used as a reference sequences. Three samples were excluded from the analysis due to low read number. UniFrac distance matrices were generated with the Jackknifed Beta Diversity workflow based on 10 distance metrics calculated using 10 subsampled OTU tables and projected using non-metric multidimensional scaling (NMDS). The number of sequences taken for each jackknifed subset was set to 85% of the sequence number within the most indigent sample (Hansen et al., 2012). Permutational Multivariate Analysis of Variance (PERMANOVA) (compare_categories.py, Qimme 1.8.0) was used to evaluate group differences based on weighted, unweighted UniFrac distance matrices. Alpha diversity measures expressed with an observed species (sequence similarity 97% OTUs) value were computed for rarefied OTU tables (25,000 reds/sample) using the alpha rarefaction workflow. Differences in alpha diversity were determined using a *t*-test-based approach employing the non-parametric (Monte Carlo) method (999 permutations) implemented in the compare alpha diversity workflow. The differences in taxa abundance were between categories were estimated with a statistic framework: analysis of composition of microbes (ANCOM) (Mandal et al., 2015) based on non-normalized OTU-table summarized to the species level.

### Statistics

Statistical analysis was performed using GraphPad Prism. Data were analyzed by one-way ANOVA or two-way ANOVA with Bonferroni's post-test or by unpaired two-tailed Student's *t*-test, as referred to for each graph in Figure legends.

## Results

### In the absence of IAV infection, antibiotic treatment of juvenile mice results in transient changes to GM composition and weight gain

**Figure 1:**
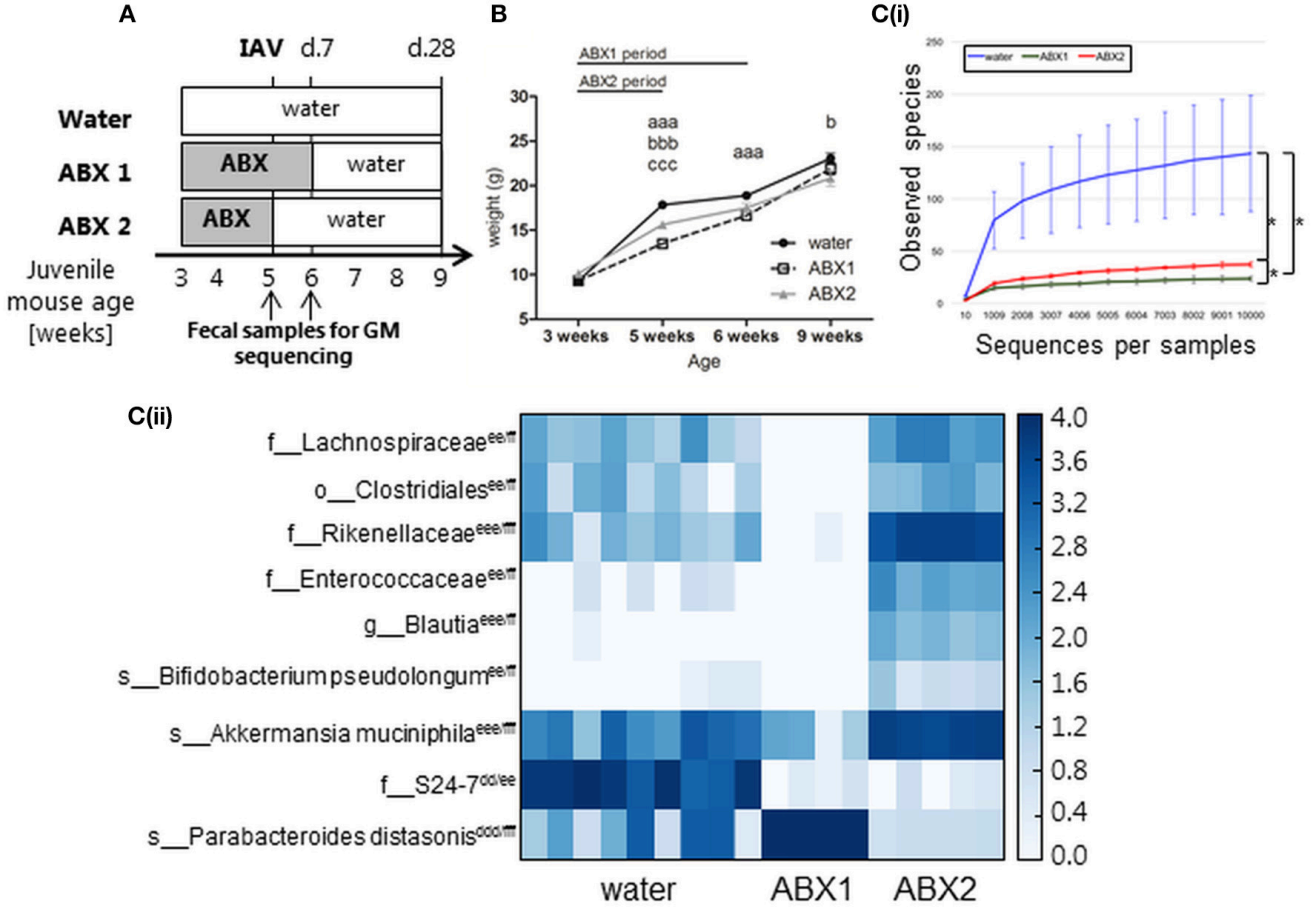
In the absence of IAV infection, antibiotic treatment of juvenile mice is associated with impaired weight gain and transient changes in GM composition. **(A)** Experimental timeline. Juvenile mice were treated with broad-spectrum antibiotics (ABX) in drinking water from the time of weaning for (i) 3 weeks (ABX1), (ii) 2 weeks followed by 1 week of normal drinking water (ABX2), or (iii) received normal drinking water throughout (water). At 5 weeks of age mice were infected via the i.n. route with 100 PFU X31 or mock-infected with an equivalent volume of virus diluent. Fecal samples were collected from ABX1, ABX2 and water groups just prior to infection (5 weeks) and 1 week post-infection (6 weeks). Animals were weighed daily and assessed for signs of disease from day 0 to 10 p.i. At day 7 or day 28 p.i., some animals were killed and analyzed for virus titres and/or immune responses to infection. **(B)** Body weights (expressed in gram) of mock-infected mice. Shown are weights from water-mock (closed circles), ABX1-mock (open squares), and ABX2-mock (gray triangles). Data for weeks 3, 5, 6 and 9 are pooled from 8 independent experiments for water-mock (*n* = 30, 80, 40, and 20 mice, respectively), ABX1-mock (*n* = 20, 40, 20, and 10 mice, respectively) and ABX2-mock (*n* = 10, 40, 20, and 10 mice, respectively) groups. The graph shows the mean ± SD of the pooled data and was analyzed using two-way ANOVA followed by Bonferroni's post-test: ^a^ = water-mock vs. ABX1-mock, ^b^ = water-mock vs. ABX2-mock, ^c^ = ABX1-mock vs. ABX2-mock with *p* < 0.001, *p* < 0.01, and *p* < 0.05 are denoted by 3, 2, or 1 letters, respectively. **[C(i)]** Rarefaction curves based on observed species index showing the alpha-diversity in GM from 6 weeks old water-mock (*n* = 9), ABX1-mock (*n* = 4), and ABX2-mock mice (*n* = 5). Data were analyzed using paired Student's *t*-test, *p < 0.05. **[C(ii)]** Heatmap depicting relative distribution of bacterial taxa in 6 weeks old water-mock, ABX1-mock and ABX2-mockand reported as significantly different between the three categories (ANCOM, *p* < 0.05). The *post-hoc* Tukey test was used to identify the source of variance. Significant paired differences are marked accordingly: ^d^ = water-mock vs. ABX1-mock ^e^ = water-mock vs. ABX2-mock, ^f^ = ABX1-mock vs. ABX2-mock with *p* < 0.001, *p* < 0.01 and *p* < 0.05 are denoted by 3, 2 or 1 letters, respectively. Abbreviations of bacterial taxonomy: *f* = family, *g* = genus, *o* = order, *s* = species.

Juvenile mice were treated with broad spectrum ABX in drinking water from the time of weaning (3 weeks old) for either (i) 3 weeks (ABX1), or (ii) 2 weeks, and subsequently returned to normal drinking water (ABX2). Control animals (water) received normal water throughout the experiment. At 5 weeks of age, mice were infected via the i.n. route with 100 PFU X31 or mock-infected with diluent, then weighed and examined for signs of disease on a daily basis. Animals were killed for analysis at 7 or 28 days p.i., corresponding to times when the animals were 6 or 9 weeks of age, respectively. Fecal samples were collected from ABX1, ABX2 and water groups prior to infection (5 weeks) and at 1 week post-infection from mock-infected mice (6 weeks). The experimental timeline is shown in Figure 1A.

First, we assessed the impact of ABX treatment on body weight and GM composition in the absence of IAV infection. The weight of all uninfected animals (water, ABX1 and ABX2) was assessed at 3 weeks of age (i.e., time of weaning when ABX treatment commenced), as well as at weeks 5, 6, and 9. Mice in all experimental groups gained weight between 3 and 9 weeks of age (Figure 1B) and no significant differences in body weight were noted between groups prior to ABX treatment at week 3. Compared to control (water) animals, ABX treatment for 2 weeks (ABX2) was associated with a significant reduction in body weight when mice were 5 and 9 weeks of age. ABX treatment for 3 weeks (ABX1) was associated with significantly reduced body weight at weeks 5 and 6 of age, but not at week 9 (Figure 1B). Thus, in the absence of infection juvenile mice show impaired weight gain during the course of ABX treatment, but this effect tends to be lost after cessation of ABX treatment.

Next we examined the impact of ABX treatment on GM composition in uninfected juvenile mice. We examined the GM composition of control (water) animals at 5 and 6 weeks of age as at this age the GM composition stabilize after perturbations due to transition to solid foods after weaning (Hirayama et al., 1995). No significant differences in the GM composition of control animals were noted between 5 and 6 weeks of age (Figure S1A). Similarly, the GM composition of mice treated with ABX for 2 weeks (assessed at 5 weeks of age) or 3 weeks (assessed at 6 weeks of age) showed no significant differences (Figure S1B). After 1 week back on regular water, ABX2 mice showed distinct GM to both ABX1 and water groups (Figure S1C). The microbial diversity expressed with observed species index differed between all three groups, with the highest diversity found in water-treated mice and the lowest diversity in ABX1-treated mice (Figure 1C(i)). Further analysis showed that nine bacterial taxa accounted for differences in GM composition between water, ABX1 and ABX2 treatment groups (Figure 1C(ii)). Not surprisingly, the microbial diversity of the ABX1 group was clearly reduced compared to the control (water) with *Parabacteroides distasonis* being dominant in the ABX1 mice. After 1 week on regular water, ABX2 mice showed increased GM diversity although the relative abundance remained quite distinct to that of control (water) animals. The relative proportions of *Akkermansia muciniphila* and Rikenellaceae and, to lesser extent, Lachnospiraceae, Clostridiales, Enterobacteriaceae, *Blautia* and *Bifidobacterium pseudolongum* increased at the expense of *Parabacteroides distasonis* (Figure 1C(ii)). Notably, the members of the S24-7 family that were relatively abundant in water mice did not recover to its original abundance after 1 week back on normal drinking water (Figure 1C(ii)). The GM composition of each juvenile mouse is depicted in (Figure S1D). Together, these data confirm that ABX treatment of juvenile mice modulates GM composition and that the GM is largely re-established, albeit with some taxa differing in relative abundance, already 1 week after cessation of ABX treatment.

### Cessation of antibiotic treatment prior to IAV infection of juvenile mice did not result in exacerbated weight loss or increased virus replication in the lungs

At 5 weeks of age, mice were infected via the i.n. route with 100 PFU of X31 or mock-infected with an equal amount of virus diluent (mock). At this time, water- and ABX2-treated mice received normal drinking water, while ABX1-treated mice remained on ABX treatment for an additional 7 days (Figure 1A). At day 7 p.i., half of the mice were killed for analysis while the other half were killed and analyzed at day 28 p.i.

**Figure 2:**
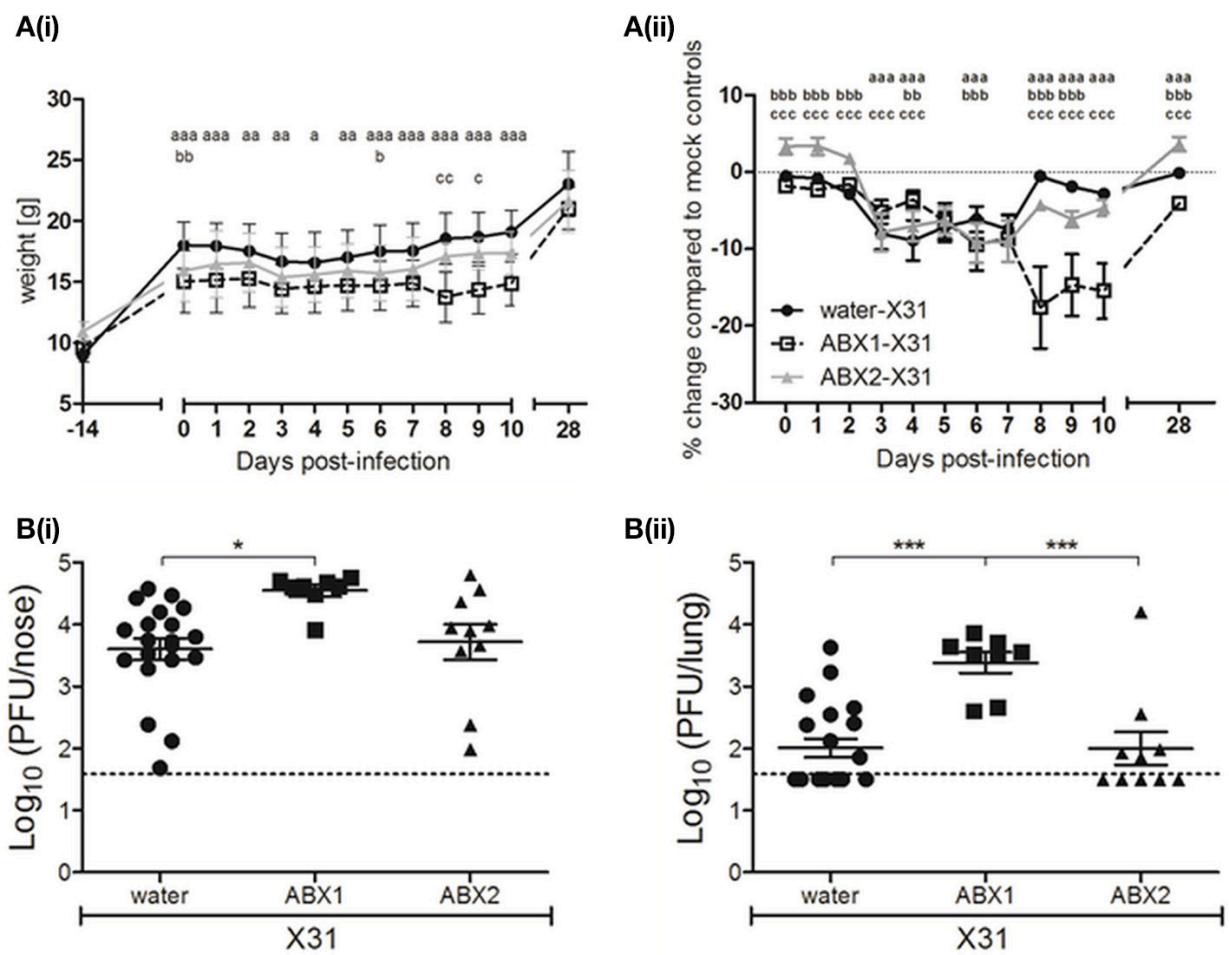
Antibiotic treatment during, but not prior to infection, exacerbates IAV-infection and disease in juvenile mice. **(A)** Body weights of X31-infected animals are shown from the day ABX treatment commenced (day−14 p.i.) to day 28 p.i. **(i)** Body weight in grams. **(ii)** Body weight as the percentage change in weight of infected mice compared to their respective mock-infected controls. Shown are water-X31 (closed circles, *n* = 40), ABX1-X31 (open squares, *n* = 20), and ABX2-X31 (gray triangles, *n* = 20) calculated relative to water-mock (*n* = 40), ABX1-mock (*n* = 20) and ABX2-mock (*n* = 20) and represent the mean ± SEM of pooled data from 8 experiments. Data were analyzed using a two-way ANOVA followed by Bonferroni's post-test. ^a^ = water-X31 vs. ABX1-X31, ^b^ = water-X31 vs. ABX2-X31, ^c^ = ABX1-X31 vs. ABX2-X31 with *p* < 0.001, *p* < 0.01, and *p* < 0.05 denoted by 3, 2 or 1 letters, respectively. **(B)** Titres of infectious virus were determined in clarified homogenates prepared from **(i)** lung and **(ii)** nasal tissues of X31-infected mice at day 7 p.i. Titers from individual mice are shown and horizontal lines depict the mean ± SEM. The dashed line indicates the cut-off value for detection of virus. Data are pooled from 4 independent experiments (water-X31, *n* = 20; ABX1-X31, *n* = 10; ABX2-X31, *n* = 10) and were analyzed using a one-way ANOVA followed by Bonferroni's post-test. ***p < 0.001, *p < 0.05.

First, we compared body weights between X31-infected mice from the different groups (water, ABX1 and ABX2) and results are presented as weight in grams (Figure 2A(i)). Compared to infected mice that received water (water-X31), ABX1-X31 mice showed reduced body weight and this was particularly pronounced at days 6–10 p.i. ABX2-X31 mice showed milder weight loss and were significantly different to water-X31 mice only on day 0 and day 6 p.i. Significant differences between ABX1-X31 and ABX2-X31 were only noted on days 8 and 9 p.i. We next examined percentage change in body weight relative to the corresponding mock-infected animals (Figure 2A(ii)) to account for the differences in weight gain observed in the uninfected mice that received water, ABX1 or ABX2 treatments (Figure 1B). In these analyses, it was clear that X31 infection was associated with mild and transient weight loss in water-X31 and ABX2-X31 groups and these animals regained body weight after day 7 p.i. However, ABX1-X31 exhibited greater and sustained weight loss compared to both water-X31 and ABX2-X31 mice, resulting in significant differences compared to both groups at days 8–10 p.i. Although animals were not weighed between days 11 and 27, ABX1-treated mice still showed a significant reduction in weight gain at day 28 p.i. compared to water-X31 and ABX2-X31 groups(Figure 2A(ii)).

Next, we assessed virus replication in the upper and lower airways of water-, ABX1- or ABX2-treated mice at day 7 p.i. Titres of infectious virus recovered from the upper respiratory tract (nose) of ABX1-treated mice were significantly higher than those from water-treated animals (Figure 2B(i)). More strikingly, virus titres in the lungs of ABX1-treated mice were significantly higher compared to both water- and ABX2-treated animals (Figure 2B(ii)). Together, these data indicate that weight loss is exacerbated and sustained in ABX1-treated mice infected with X31 and this correlated with enhanced virus replication, particularly in the lung. In contrast, cessation of ABX treatment at the time of infection (ABX2-X31), resulted in weight loss and virus replication that was similar to that observed in X31-infected mice that received normal drinking water (water-X31).

### Cessation of antibiotic treatment prior to IAV infection of juvenile mice does not impair IAV-specific T cell or humoral responses

T lymphocytes play integral roles in recovery from primary influenza infection as well as following subsequent re-exposure to virus. Naïve virus-specific T cells are activated by lung DCs in the medLNs where the T cells undergo differentiation and proliferation (Legge and Braciale, 2003; Lawrence and Braciale, 2004). CD4^+^ and CD8^+^ T effector cells then migrate to the lung where they act in concert to clear the virus (Swain et al., 2004). A proportion of CD8^+^ T cells differentiate into T effector memory (T_EM_) cells which predominantly localize to the lung while others differentiate into T central memory (T_CM_) cells which migrate to and persist in the spleen and medLNs, enabling a fast and efficient response to a secondary IAV infection (Swain et al., 2004).

**Figure 3:**
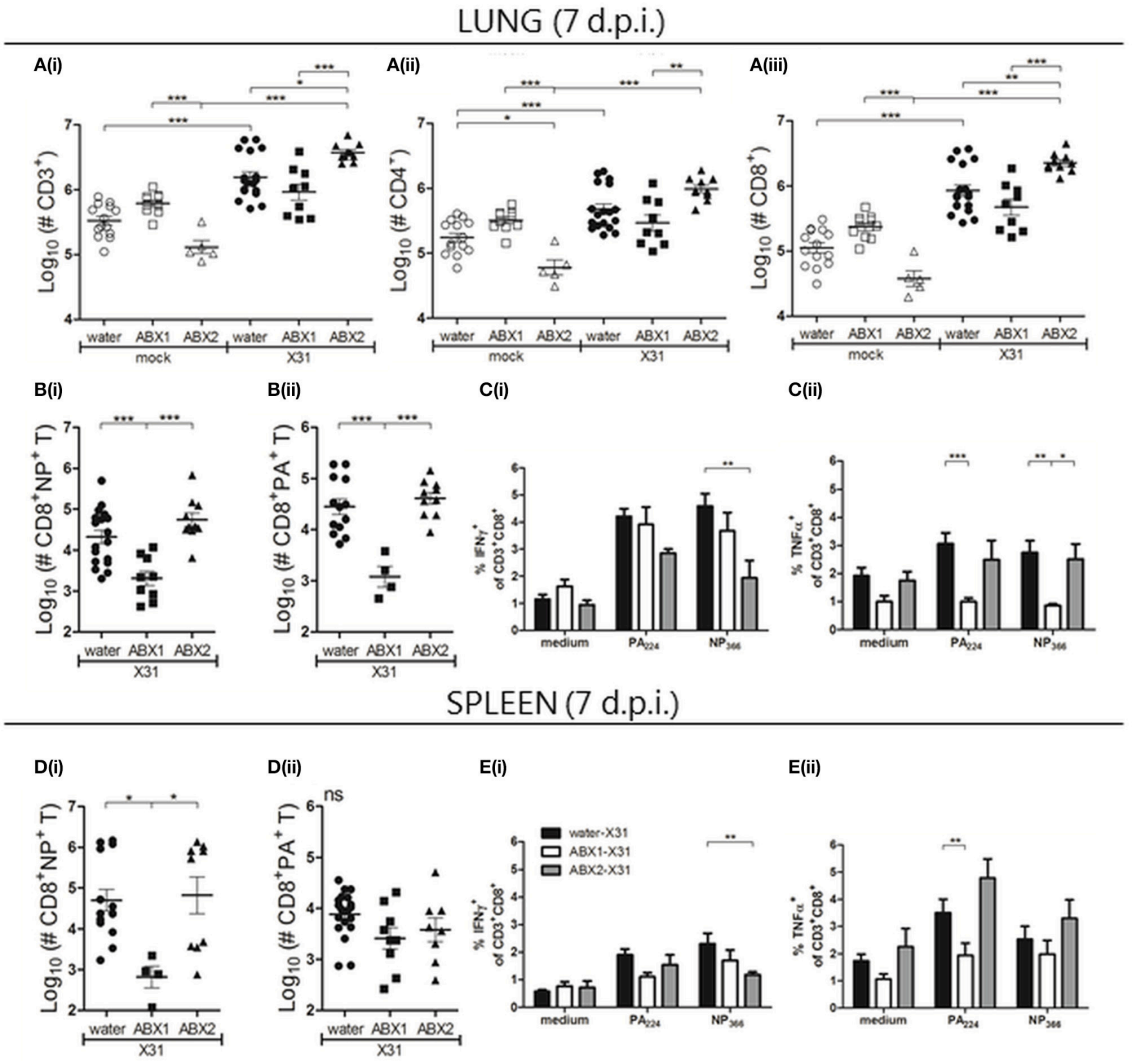
Antibiotic treatment prior to and during, but not prior to infection, impairs IAV-induced immediate lymphocyte responses in lung and spleen. At day 7 p.i., single cell suspensions prepared from the lungs **(A–C)** and spleen **(D,E)** were analyzed by flow cytometry. **(A)** Numbers of **(i)** total CD3^+^, **(ii)** CD3^+^CD4^+^ and **(iii)** CD3^+^CD8^+^ lymphocytes. **(B/D)** Numbers of **(i)** NP- and **(ii)** PA-specific CD8^+^ T cells. For A, B and D, results from individual animals are shown with the horizontal lines depicting the mean ± SD. Data represent results from water-mock (*n* = 15), ABX1-mock (*n* = 10), ABX2-mock (*n* = 5), water-X31 (*n* = 20), ABX1-X31 (*n* = 10) and ABX2-X31 (*n* = 10). **(C/E)** Intracellular cytokine staining was performed following incubation of **(C)** lung and **(E)** spleen cell suspensions with PA/NP-specific peptides, or with medium alone. The percentage of CD8^+^ T lymphocytes staining positive for intracellular IFNγ or TNFα are shown from water-X31 (black bars, *n* = 15), ABX1-X31 (white bars, *n* = 9) and ABX2-X31 (gray bars, *n* = 5) mice. Data are shown as the mean ± SD. For statistical analyses, data were analyzed using either one-way ANOVA **(A,B,D)** or two-way ANOVA **(C,E)**, followed by Bonferroni's post-test. ***p < 0.001, **p < 0.01, *p < 0.05.

Flow cytometry was used to assess numbers and proportions of lymphocytes in the lung, spleen and medLN at day 7 p.i., a time corresponding to the early influx of T cells (gated on CD3) into the airways (Flynn et al., 1998; Tate et al., 2012). In mock-infected animals there was a tendency for numbers of total CD3^+^ (Figure 3A(i)), CD4^+^ (Figure 3A(ii)) or CD8^+^ (Figure 3A(iii)) to be lower in lungs of ABX2-mock animals compared to cells from mock-infected water or ABX1 groups. Numbers of CD3^+^, CD4^+^, and CD8^+^ lymphocytes increased significantly between mock and X31-infected animals from water- and ABX2-treated groups at day 7 p.i. but were not significantly different between mock- and X31-infected animals from the ABX1 group (Figure 3A). Moreover, numbers of CD3^+^, CD4^+^, and CD8^+^ recovered from lungs of ABX1-X31 mice were significantly lower than those recovered from ABX2-X31 mice at day 7 p.i. (Figure 3A).

Next, we analyzed numbers of virus-specific CD8^+^ T cells in the lungs using PA_224_- or NP_366_-specific tetramers. Numbers of NP- (Figure 3B(i)) and PA-specific CD8^+^ T cells (Figure 3B(ii)) in the lung were significantly reduced in ABX1-treated mice compared to either water-X31 or ABX2-X31 mice. No significant differences were noted in numbers of NP- and PA-specific CD8^+^ T cells between water-X31 and ABX2-X31 groups. We also examined levels of intracellular IFNγ, tumor necrosis factor (TNF) α and interleukin (IL)-2 expressed by NP- and PA-specific CD8^+^ T cells in the lung (Figure 3C). First, we observed a lower proportion of CD8^+^NP^+^ T cells from ABX2-treated mice that produced IFNγ compared to cells from water-treated animals (Figure 3C(i)). Furthermore, PA-specific CD8^+^ T cells from ABX1-treated mice produced less TNFα than cells from either water- or ABX2-treated animals (Figure 3C(ii)) and levels of IL-2 were similar to medium-only controls in all groups (Figure S4) Together, these data indicate that ABX treatment of juvenile mice prior to and during X31-infection reduced the number of lymphocytes in the lungs at day 7 p.i., including virus-specific CD8^+^ T cells. Treatment for 2 weeks prior to, but not during IAV infection (ABX2), did not alter the early recruitment of lymphocytes to the lungs. However, both ABX treatments resulted in differential expression patterns of some intracellular cytokines in virus-specific CD8^+^ T cells.

In the spleen, we detected no significant differences in numbers of splenic CD3^+^ cells, or CD4^+^ or CD8^+^ T cells between mock- or IAV-infected mice in each of their respective groups (water, ABX1 or ABX2; Figure S4). However, numbers of NP-specific CD8^+^ T cells were reduced in IAV-infected ABX1 mice compared to water- or ABX-treated groups (Figure 3D(i)), although numbers of PA-specific CD8^+^ T cells were not different between infected groups (Figure 3D(ii)). As in the lung, we observed that a lower proportion of CD8^+^NP^+^ T cells from ABX2-treated mice that produced intracellular IFNγ compared to cells from water-treated animals (Figure 3E(i)). Furthermore, intracellular TNFα production was reduced in both PA- and NP-specific CD8^+^ T cells from ABX1-treated mice compared to cells from either water- or ABX2-treated animals (Figure 3E(ii)). Of note, we did not see any differences in numbers of lymphocytes (CD3^+^, CD4^+^, and CD8^+^ T cells, as well as NP-/PA-CD8^+^ cells,), or in the percentage of CD8^+^ T cells producing intracellular cytokines following stimulation with viral peptides, in medLNs between the three X31-infected groups (data not shown).

**Figure 4:**
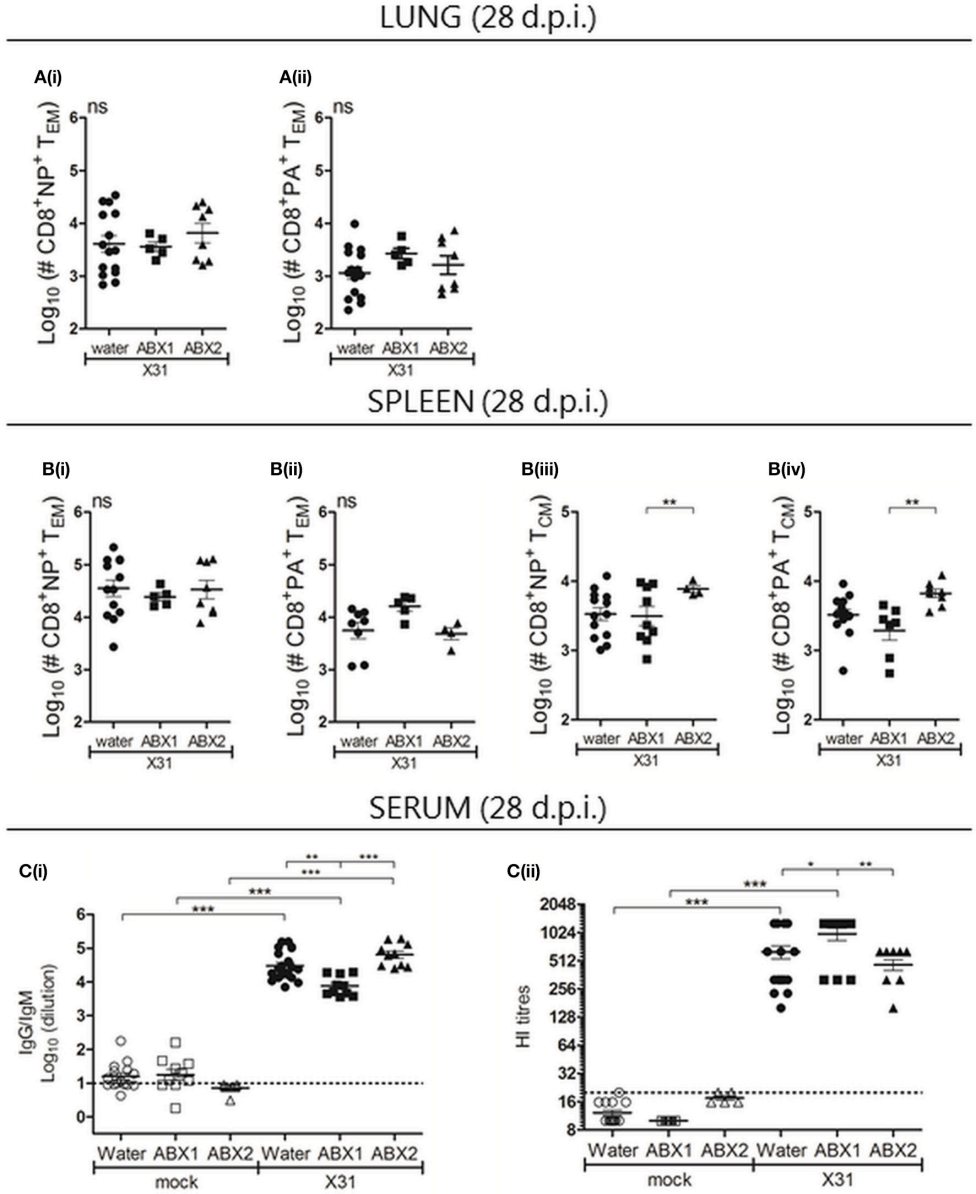
Assessment of CD8 T cell and humoral immune responses in mice that received water-, ABX1 and ABX2 treatments at day 28 after infection with IAV. Single cell suspensions prepared from the **(A)** lungs and **(B)** spleen were analyzed by flow cytometry. Numbers of **(i)** NP- and **(ii)** PA-specific CD8^+^ T_EM_ in the lung and spleen are shown. In the spleen, numbers of **(iii)** NP- and **(iv)** PA-specific CD8^+^ T_CM_ were also determined. Results from individual animals are shown with horizontal lines depicting the mean ± SD. Data represent results from water-X31 (*n* = 20), ABX1-X31 (*n* = 10) and ABX2-X31 (*n* = 10) and were analyzed using one-way ANOVA followed by Bonferroni's post-test. **(C)** Titres of X31 specific antibodies were determined by ELISA **[C(i)]** or by HI assay **[C(ii)]**. For **(C)**, titres for individual mice are shown and horizontal lines depict the mean ± SD. The detection limit of each assay is indicated by the dashed line. Data represent results from water-mock (*n* = 15), ABX1-mock (*n* = 10) and ABX2-mock (*n* = 5), water-X31 (*n* = 20), ABX1-X31 (*n* = 10), and ABX2-X31 (*n* = 10) and were analyzed using one-way ANOVA followed by Bonferroni's post-test. ***p < 0.001, **p < 0.01, *p < 0.05.

Next, CD8^+^ T cell and humoral immune responses were assessed at day 28 p.i. No significant differences were observed between groups in numbers of CD3^+^, CD4^+^, CD8^+^ cells in the lung and spleen (Figure S4). Similarly, numbers of (i) NP- or PA-specific CD8^+^ T_EM_ cells (CD44^+^CD62L^−^) in the lung (Figures 4A(i/ii)) and spleen (Figures 4B(i/ii)) were not different between groups, although numbers of NP- and PA-specific CD8^+^ T_CM_ cells (CD44^+^CD62L^+^) were significantly higher in ABX2-treated compared to ABX1-treated animals (Figures 4B(iii/iv)). We next used ELISA and HI assays to assess humoral responses to X31 infection at day 28 p.i. Compared to the relevant mock-infected controls, infection was associated with significantly enhanced titres of virus-specific IgG/IgM by ELISA (Figure 4C(i)), as well as enhanced HI titres (Figure 4C(ii)). At day 28 p.i., ABX1-treated mice showed significantly lower titres of virus-specific antibody by ELISA compared to both water- and ABX2-treated mice. Despite this, ABX1-X31 mice had enhanced serum HI titres at this time compared to both water-X31 and ABX2-X31 mice. The CD8^+^ T cells and antibody memory response to influenza is not affected by ABX treatment.

### Antibiotic treatment of pregnant dams leads to altered GM composition of dams and impaired postnatal weight gain in pups

**Figure 5:**
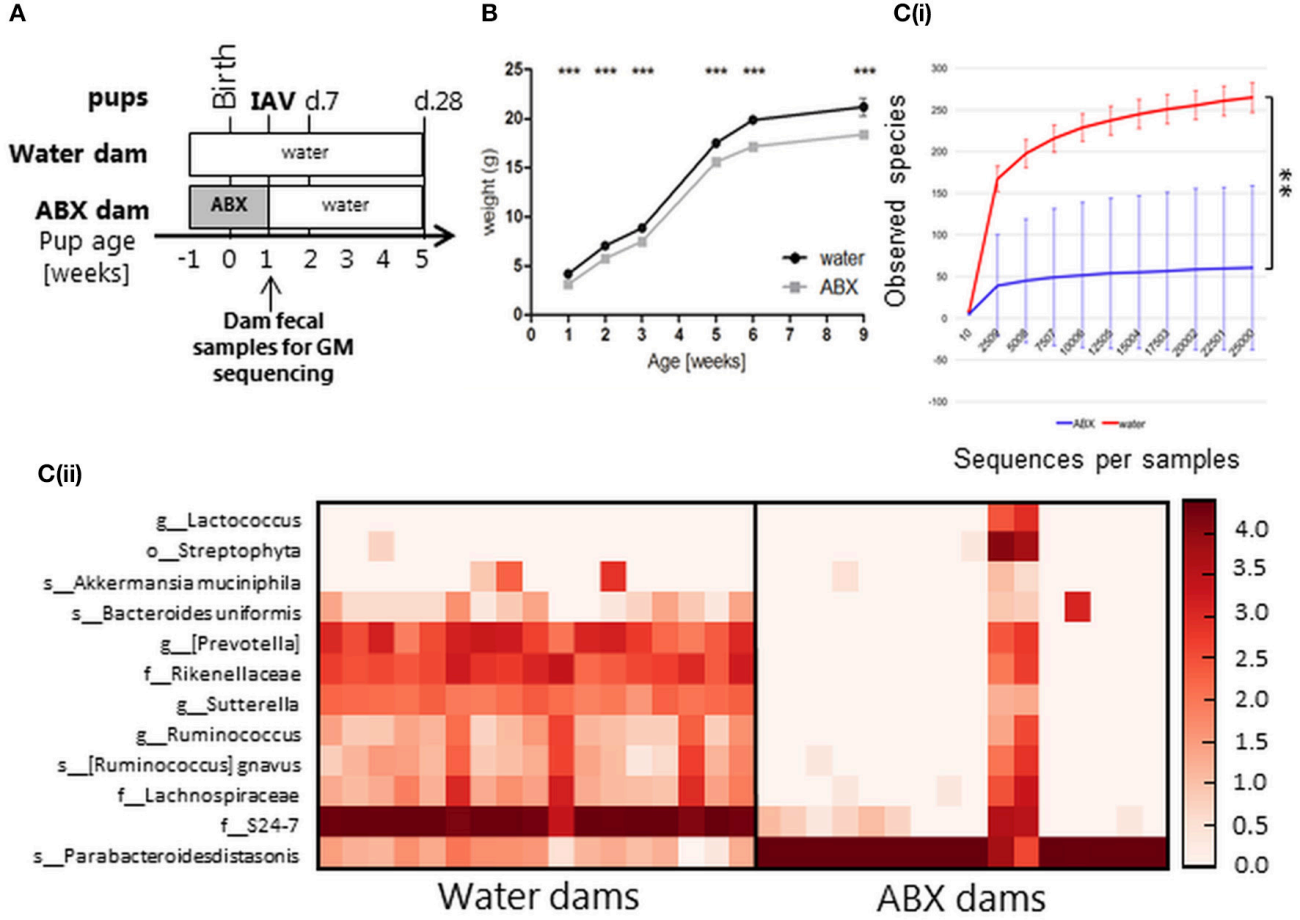
Antibiotic treatment of pregnant dams changes their GM composition at time of birth, which leads to impaired post-natal weight gain in pups into adulthood and changes in pup GM 5 weeks post-partum. **(A)** Experimental timeline. Pregnant dams were treated with ABX in drinking water for 2 weeks from time of confirmed pregnancy (1 week pre-partum, ABX) followed by normal drinking water or received normal drinking water throughout (water). At 1 week of age pups were infected via the i.n. route with 20 PFU of X31 or mock-infected with an equivalent volume of virus diluent. Animals were weighed daily and assessed for signs of disease from day−1 to day 10 p.i. At day 7 or day 28 p.i., some animals were killed and analyzed for virus titres and/or immune responses to infection. Fecal samples were collected from dams in both groups immediately after cessation of ABX treatment. **(B)** Weight gain (expressed in gram) of mock-infected pups. Shown are weights for water-mock (black circles), ABX-mock (gray squares). Data for weeks 1, 2, 3, 5, 6, and 9 are pooled from 12 independent experiments with pups derived from water mock (*n* = 72, 80, 41, 36, 27, 8, respectively) or ABX-mock (*n* = 75, 89, 37, 42, 31, 6, respectively) treated dams. The graph shows the mean ± SEM of pooled data and was analyzed using 2-way ANOVA followed by Bonferroni's post-test. ***p < 0.001. **[C(i)]** Rarefaction curves based on observed species index showing the alpha-diversity in GM from dams ± 2 weeks of ABX treatment (water, green, *n* = 18; ABX, blue, *n* = 18). Data were analyzed using Student's *t* test, **p < 0.01. **[C(ii)]** Heatmap depicting the relative distribution of bacterial taxa reported as significantly different between the water dam and ABX dam (ANCOM, *p* < 0.05). Abbreviations of bacterial taxonomy: *f* = family, *g* = genus, *o* = order, *s* = species.

While perinatal ABX treatment has been associated with compromised CD8^+^ T cells responses and enhanced morbidity and mortality in pups infected with vaccinia virus (VV) (Gonzalez-Perez et al., 2016), its effects on IAV infection of pups has not been determined. Therefore, pregnant dams were treated with the same ABX cocktail as juvenile mice from 1 week pre-partum to 1 week post-partum (see Figure 5A for experimental timeline). We did not observe any differences in litter size from water- or ABX-treated dams (Figure S5). After birth, pups derived from dams that did or did not receive ABX treatment were monitored and body weight assessed at 1, 2, 3, 5, 6, and 9 weeks of age in the absence of IAV infection (Figure 5B). While overall body weight of pups from both water- or ABX-treated dams increased at each time point, animals derived from ABX-treated dams showed significantly reduced body weight at all time points compared to those from dams that received normal drinking water.

Fecal samples collected from dams on the day that ABX treatment ceased (i.e., when pups reached 7 days of age) were assessed to determine GM composition. In rats, it has previously been shown that the GM composition of pups at 2 weeks post-partum resembled that of their mothers treated with ABX from days 3 to 4 pre-partum (Fåk et al., 2008). We therefore assumed that dam GM composition at the end of perinatal ABX treatment (7 days post-partum) would be indicative of GM composition in pups at the time of infection. The microbial diversity expressed with observed species index differed between water-treated dams and ABX-treated dams (Figure 5C(i)). The abundance and/or prevalence of 12 bacterial taxa accounted for the differences between the water dams and ABX dams (Figure 5C(ii)). The GM diversity of ABX dams was significantly reduced, in favor of *Parabacteroides distasonis* as also observed for the juvenile mice (Figure 1C). This correlates with the sustained delay in weight gain observed in ABX pups between 1 and 9 weeks of age (Figure 5B). Notably, two animals from the ABX dam groups responded poorly to ABX treatment as evidenced by a broader representation of different bacterial taxa (Figure 5C(ii)). The GM composition of each dam is depicted in Figure S2B.

### Perinatal antibiotic treatment does not have major impacts on disease severity during the acute phase of X31 infection, but was associated with a major delay in long-term weight gain in X31-infected pups

In preliminary experiments, 7 day old pups from water- or ABX-treated dams infected via the i.n. route with 100 PFU of X31 exhibited clinical signs that necessitated euthanasia (data not shown). Therefore, we modified our experiments such that 7 day old pups were infected with 20 PFU of X31 and were monitored and weighed daily until day 10 p.i, and then again at day 28 p.i.

**Figure 6:**
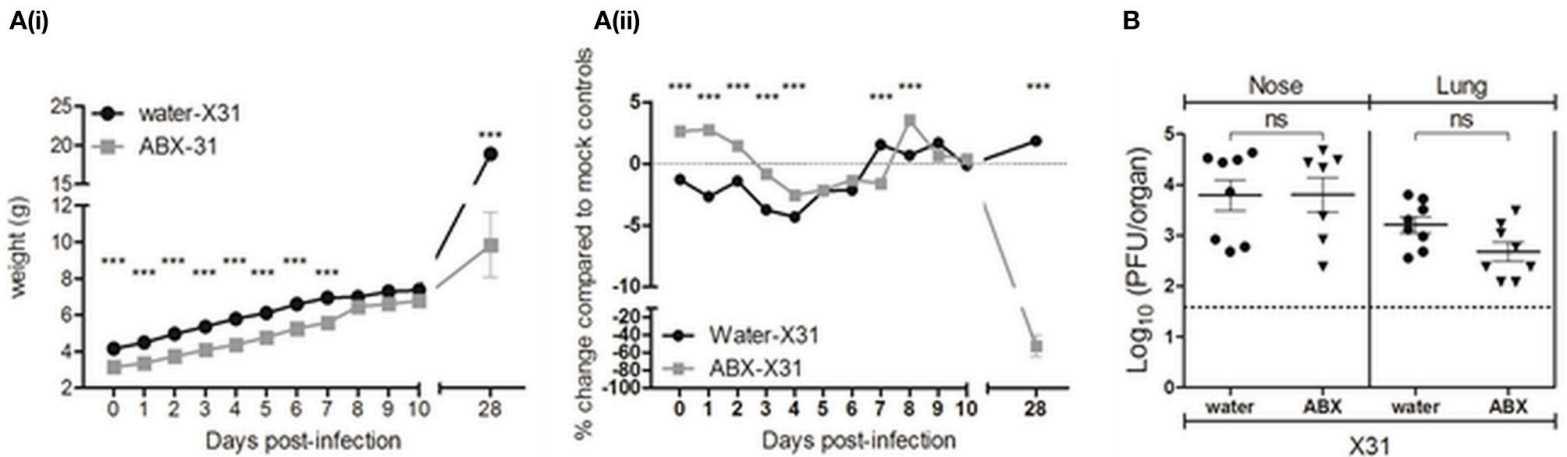
Perinatal antibiotic treatment does not have major impacts on the acute phase of IAV infection in pups, but does result in significant impairment of postnatal weight gain. **(A)** Body weights of X31-infected pups are shown from the day of infection (day 0 p.i.) to day 28 p.i. **(i)** Body weight in grams. **(ii)** Body weight as the percentage change in weight (in grams) of infected mice compared to their respective mock-infected controls. Shown are water-X31 (black circles, *n* = 7–44 on any particular day) and ABX-X31 (gray squares, *n* = 4–40 on any particular day) calculated relative to water-mock (*n* = 7–28 on any particular day) and ABX-mock (*n* = 13–34 on any particular day). Data are shown the mean ± SEM of pooled data from 6 experiments and were analyzed using a two-way ANOVA followed by Bonferroni's post-test. ***p < 0.001. **(B)** Titres of infectious virus were determined in clarified homogenates prepared from lung and nasal tissues of X31-infected pups at day 7 p.i. Titers from individual mice are shown and horizontal lines depict the mean ± SEM. The dashed line indicates the cut-off value for detection of virus. Data are pooled from 2 independent experiments (water-X31, *n* = 8 and ABX-X31, *n* = 7) and were analyzed using a Student's *t*-test. *ns*, not significant.

At time of infection (day 0), pups from ABX-treated dams weighed significantly less than pups from water-treated dams (ABX-X31 3.13 ± 0.48 g vs. water-X31 4.17 ± 0.49 g) (Figure 6A(i)). Body weights of ABX-X31 pups remained significantly lower than water-X31 pups during the first week post-infection and were also significantly reduced at day 28 p.i. Thus, while ABX-X31 mice continued to gain body weight following IAV infection, they did so at a markedly reduced rate relative to water-X31 controls. This was particularly apparent when we examined the percentage change in body weight compared to the corresponding mock-infected controls, to adjust for differences in weight gain observed between uninfected animals derived from dams that received ABX or normal drinking water (Figure 6A(ii)). While X31 infection induced mild and transient weight loss in animals that did or did not receive perinatal ABX treatment, by day 10 p.i. all animals had recovered weight relative to relevant mock-infected controls. However, for animals derived from ABX-treated dams, X31-infected animals exhibited a profound reduction in body weight relative to mock-infected controls at day 28 p.i. and this was not observed in animals derived from dams that received normal drinking water. These findings indicate that X31 infection induces a transient weight loss in pups from water- or ABX-treated dams, however infection early in life has a major impact on weight gain in animals derived from ABX-treated dams.

Next, we determined titres of infectious virus in both the upper (nose) and lower (lungs) respiratory tract at day 7 p.i. While virus could be detected at both sites, there were no significant differences in titres between pups derived from water- or ABX-treated dams (Figure 6B). In an independent experiment, we confirmed that infectious virus had been cleared from both sites by day 14 p.i. (data not shown). These findings indicate that persistent virus replication did not contribute to the delayed gains in percentage body weight observed in ABX-X31 mice relative to ABX-mock controls (Figure 6A(ii)). Thus, during the acute phase of X31 infection (days 1–10 p.i.) pups derived from water- or ABX-treated dams did not show major differences in body weight or virus replication in the airways. Relative to ABX-mock controls, X31 infection resulted in markedly reduced gains in body weight between days 10 and 28 p.i. suggesting that IAV infection in early life somehow slowed the rate of weight gain in pups, even after clearance of infectious virus from the airways.

### Perinatal antibiotic treatment does not result in major changes of acute or memory CD8^+^ T cell responses, or in humoral immunity, in pups following X31 infection

**Figure 7:**
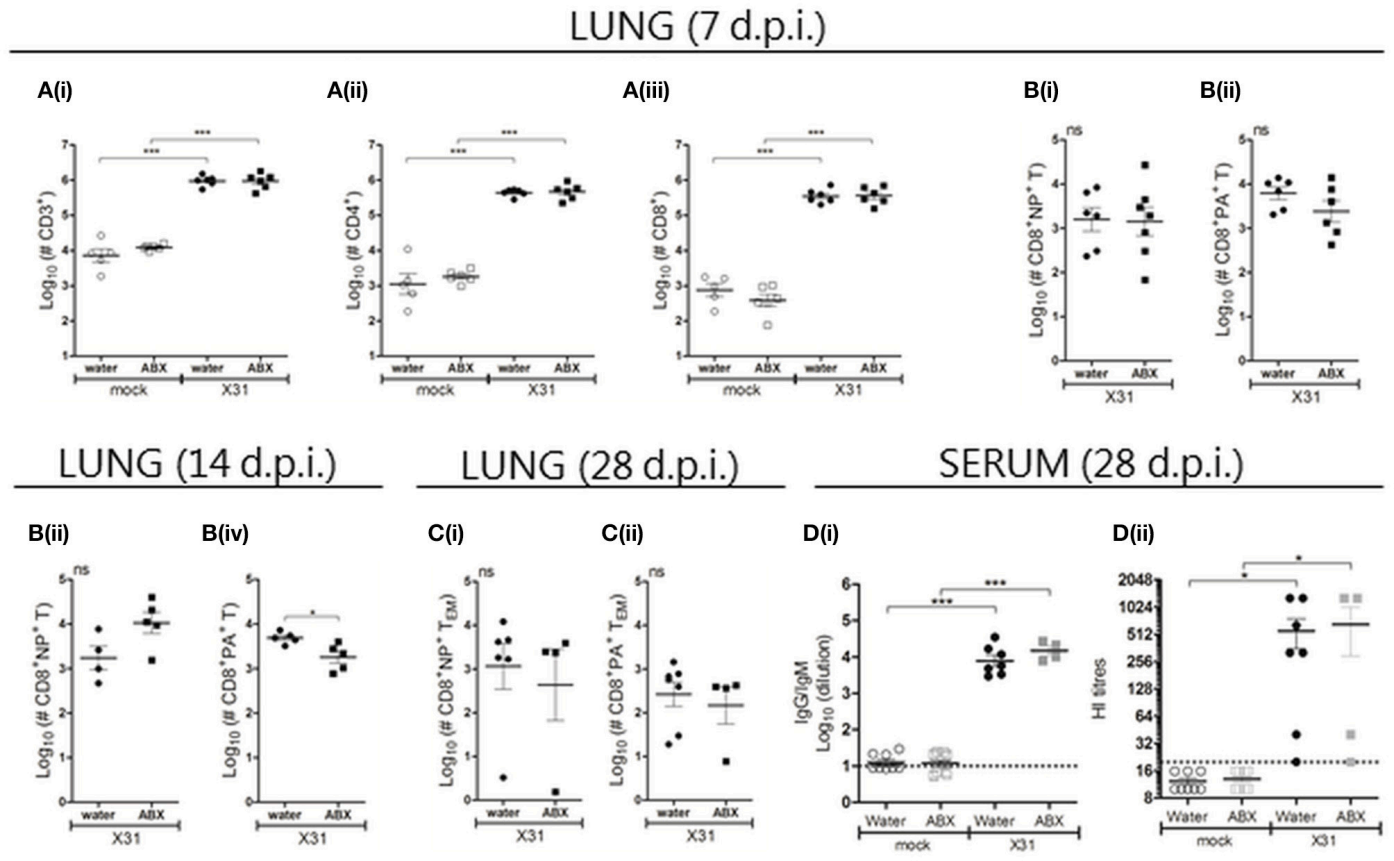
Perinatal antibiotic treatment does not result in major changes of acute or memory CD8^+^ T cell responses, or in humoral immunity, in pups following X31 infection. At day 7 **[A/B(i/ii)]**, 14 **[B(iii/iv)]** and 28 **(C)** p.i., single cell suspensions prepared from the lungs were analyzed by flow cytometry. **(A)** Numbers of lung lymphocytes. **(B)** Numbers of NP- and PA-specific CD8^+^ T cells recovered from the lung at **(i/ii)** day 7, **(iii/iv)** day 14 and **(C)** day 28 after infection. For **(A–C)**, results from individual animals are shown with the horizontal lines depicting the mean ± SD. Data represent results from day 7, 14, and 28 for water-mock (*n* = 5, 10, and 8, respectively), ABX-mock (*n* = 6, 12, and 13, respectively), water-X31 (*n* = 11, 8, and 7, respectively) and ABX-X31 (*n* = 9, 9, and 4, respectively). Data were analyzed using one-way ANOVA followed by Bonferroni's post-test **(A)** or Student's *t*-test **(B/C)**. ***p < 0.001, *p < 0.05, *ns* = no significance. **(D)** Serum samples collected from X31- or mock-infected animals at day 28 p.i. were **(i)** examined by ELISA to determine overall titres of X31-specific antibodies and **(ii)** examined by HI assay. Titres for individual mice are shown and the horizontal lines depict the mean ± SD. The detection limit of each assay is indicated by the dashed line. Data represent results from water-mock (*n* = 8), ABX-mock (*n* = 13), water-X31 (*n* = 7), and ABX-X31 (*n* = 4) and were analyzed using one-way ANOVA followed by Bonferroni's post-test. ***p < 0.001, *p < 0.05.

Although perinatal ABX treatment did not affect viral replication in the respiratory tract of pups at day 7 p.i., differences in gain of body weight may be associated with differences in immune responses between pups born to ABX- or water-treated dams. Given the young age of these animals when infected with X31, T lymphocyte responses were assessed at days 7 and 14 p.i. in case there was a delay in generation of effector T cell responses (Zens et al., 2017). At day 7 p.i. increased numbers of CD3^+^ lymphocytes, including CD4^+^ and CD8^+^ lymphocytes, were recovered from the lungs of infected animals compared to corresponding mock-infected animals, but no effect of ABX treatment was observed (Figure 7A). Closer examination of the CD8^+^ T cell compartment at day 7 p.i. revealed that perinatal ABX-treatment was not associated with significant differences in numbers of NP- or PA-specific CD8^+^ T cells (Figures 7B(i/ii), nor their capacity to produce intracellular cytokines following stimulation of total lung cells with NP or PA-specific peptides (Figure S5). At day 14 p.i., no differences were detected in numbers of total lung CD3^+^, CD4^+^ or CD8^+^ T lymphocytes between pups derived from water- or ABX-treated dams (Figure S5). However, numbers of PA- but not NP-specific CD8^+^ T cells in the lung were reduced in ABX-X31 compared to water-X31 pups (Figures 7B(iii/iv)). In additional experiments, pups derived from water- or ABX-treated dams were infected at 2 or 5 weeks of age with 100 PFU X31 and the lymphocyte composition in the lungs was assessed 7 days later. In these experiments, perinatal ABX treatment did not affect numbers of CD3^+^, CD4^+^ or CD8^+^ T lymphocytes in the lungs, nor did it alter numbers of NP- or PA-specific CD8^+^ T cells (data not shown). At day 7 p.i. no significant differences were noted in the splenic T lymphocyte compartment of pups derived from water- or ABX-treated dams, including in numbers of splenic NP- and PA-specific CD8^+^ T cells (data not shown).

We also investigated the lymphocyte composition in the lungs and spleen at day 28 p.i., but did not observe any significant differences between water-X31 and ABX-X31 animals in terms of (i) numbers of CD3^+^, CD4^+^ or CD8^+^ T lymphocytes at either site (data not shown), (ii) numbers of NP- or PA-specific CD8^+^ T_EM_ cells in lung (Figure 7C) and NP- or PA-specific CD8^+^ T_EM_ and T_CM_ cells in spleen (data not shown), or (iii) the ability of lung or spleen cells to produce intracellular cytokines following NP/PA peptide stimulation (data not shown). Furthermore, no significant differences were noted in titres of virus-specific IgG/IgM or HI antibodies between X31-infected animals from water- or ABX-treated dams (Figure 7D). Thus, ABX treatment of dams in the perinatal period does not impair the pups' capacity to mount an effective immune response to IAV.

## Discussion

Previous studies demonstrated that oral ABX treatment prior to and during IAV infection of adult mice profoundly altered GM and was associated with increased susceptibility and impaired immunity to IAV (Ichinohe et al., 2011; Abt et al., 2012). Our studies demonstrate that ABX treatment significantly changed GM composition in juvenile mice and in ABX-treated dams. However, if ABX treatment ceased at the time of infection, juvenile mice did not show enhanced susceptibility to IAV, nor were major differences detected in cellular and humoral adaptive antiviral immunity. Similarly, perinatal ABX treatment prior to infection of neonatal pups did not result in major differences in the susceptibility or immunity to the subsequent IAV infection. Given the widespread use of ABX, these findings have relevance to our understanding of how prior ABX treatment in early life may impact susceptibility and immunity to subsequent infection with influenza infections.

In the juvenile mouse model, the GM composition in control mice that received normal drinking water did not change significantly between 5 to 6 weeks of age, suggesting that GM stabilization had occurred. Compared to control (water) mice, ABX treatment from the day of weaning for 3 weeks (ABX1) reduced the GM diversity by targeting most bacteria except for *Parabacteroides distasonis*, which were therefore enriched in ABX-treated mice. This is consistent with findings that *Parabacteroides distasonis* isolated from children was shown to be highly resistant to most ABX (Avelar et al., 2001; Nakano et al., 2011). However, if ABX treatment of juvenile mice ceased after 2 weeks and animals received normal drinking water for 1 week (ABX2), the GM composition was markedly different to that of ABX1, but more similar to that of water mice. Only the phyla S24-7 did not re-establish in GMs of ABX2 mice. In a previous study, the GM had been restored within 1 week post-ABX treatment although its composition was slightly different to that of untreated control mice (Croswell et al., 2009). Direct ABX-treatment of juvenile mice reduced gains in body weight, but only during the actual time of ABX treatment. We speculate that commencement of ABX treatment at the time of weaning may interfere with a dynamic period of GM and body development (Hirayama et al., 1995), and this may result in the impaired weight gains observed in our study.

Perinatal ABX treatment of pregnant dams induced similar changes in GM composition to those observed in juvenile mice treated with ABX1. Both groups were characterized by clear reduction in relative abundance of most bacterial taxa except for *Parabacteroides distasonis*. The GM composition was dominated by members of the S24-7 family. We hypothesized that treating dams with oral ABX pre- to post-partum would change the diversity of colonizing bacteria in pups at birth. Perinatal ABX treatment resulted in reduced post-partum weight gain in pups for up to 9 weeks of age, indicating that modulating GM colonization in this way had lasting effects on the pups. This is in line with our findings from the juvenile mice where a short course of oral ABX altered the GM composition and reduced weight gain as well. The specific factors underlying the reduced weight gain in juvenile mice or pups following ABX treatment remain unclear. For example, altered metabolism as the result of a different GM composition in juveniles or pups could affect nutrient uptake, resulting in delayed weight gain. Further studies would be required to clarify this. However, impaired postnatal gain in weight of pups contrasts previous studies which reported that perinatal ABX treatment was not associated with significant differences in post-partum weight gain of pups (Deshmukh et al., 2014; Gonzalez-Perez et al., 2016). Differences in environmental bacteria at different facilities are likely to be one factor contributing to these discordant findings. For example, a higher level of hygiene in one housing facility appeared to modulate susceptibility of pups derived from ABX-treated dams to VV infection (Gonzalez-Perez et al., 2016), suggesting that exposure to particular environmental bacteria can impact susceptibility of neonatal pups and therefore might impact other characteristics such as weight gain and growth.

The changes in GM noted in ABX1-treated juvenile mice were associated with enhanced susceptibility to X31 infection and impaired acute CD8^+^ T cell and humoral responses. This confirmed previous findings in adult mice where continued ABX treatment prior to and during the acute phase of infection with the mouse-adapted PR8 strain (Ichinohe et al., 2011; Abt et al., 2012) or X31 (Abt et al., 2012) was associated with enhanced weight loss (Abt et al., 2012), increased virus titres in lung (Ichinohe et al., 2011; Abt et al., 2012), and reduced numbers of influenza-specific CD8^+^ T cells in bronchoalveolar lavage, medLNs, spleen (Abt et al., 2012) and lung (Ichinohe et al., 2011; Abt et al., 2012). Abt et al. also demonstrated a reduced ability of virus-specific memory CD8^+^ T cells to produce multiple cytokines at day 31 after infection with PR8 (Abt et al., 2012). While we detected reduced numbers of virus-specific CD8^+^ T cells in the early acute phase of infection this did not translate into any detectable impairment of the early memory or humoral responses at day 28 p.i. However, while day ~30 p.i. represents a useful time to examine early memory responses to primary IAV infection in adult mice (Wu et al., 2014), the kinetics and magnitude of these responses might be different in juvenile mice, requiring more thorough characterization.

A number of factors may contribute to our findings that ABX1-treatment of juvenile mice was associated with enhanced viral replication and reduced CD8^+^ T cells during the acute phase of infection. Other studies examining the impact of ABX treatment of IAV infection in adult mice have demonstrated that GM contributes to the expression of antiviral genes in macrophages in the lung (Abt et al., 2012) and to the migration of DC from infected lungs to medLNs where they play a critical role in initiating CD8^+^ T cell responses (Ichinohe et al., 2011). However, our study did not find any changes in numbers of virus-specific lymphocytes in medLNs, suggesting that migration and antigen-presentation by DCs were not impaired by ABX1 treatment. Of interest, these studies showed a positive effect of toll-like receptor (TLR)-agonists administered rectally or i.n. at time of PR8 infection and a study showed a beneficial effect of orally administered *Lactobacillus gasseri* SBT2055 on PR8-induced morbidity and immune response (Nakayama et al., 2014). In our study, we demonstrate that re-colonization following cessation of ABX treatment rapidly gives rise to enhanced GM diversity (i.e., ABX2) and this, in turn, is associated with the restoration of effective immunity to IAV infection.

After challenge with IAV at 7 days of age, we did not observe any major differences in pups derived from water- or ABX-treated dams in regard to weight loss, virus replication or acute CD8^+^ T cell responses. By day 14 p.i., virus had been cleared from all groups and differences in CD8^+^ T cell responses were not observed at day 14 or 28 p.i., nor were any differences detected in humoral responses at day 28. These findings contrast studies by Gonzalez-Perez et al., who reported that infant mice born to perinatal ABX-treated dams were significantly more susceptible to VV infection (Gonzalez-Perez et al., 2016; Gonzalez-Perez and Lamousé-Smith, 2017). However, many factors could account for these discrepancies. For example, in our study pups received 20 PFU of X31 via the i.n. route compared to ~10^5^ PFU of recombinant vaccinia-OVA via the intra-peritoneal route (Gonzalez-Perez et al., 2016). In addition, we infected pups at 7 days of age compared to 15–20 days in the VV study (Gonzalez-Perez et al., 2016) and it is well established that T cell responses can be compromised in very young animals. For example, in the absence of infection 1 week old mice were reported to have significantly reduced numbers of splenic CD4^+^ and CD8^+^ cells, as well as lung CD8^+^ cells, compared to mice that were 2 weeks or older (Zens et al., 2017). To account for this and investigate any lasting impacts of ABX treatment on CD8^+^ T cells, pups derived from water- or ABX-treated dams when challenged at 2 or 5 weeks of age however we did not detect any differences in splenic or lung lymphocyte populations when assessed at 7 days p.i after either challenge. Finally, the ABX treatment period (1 week pre-partum to 1 week post-partum) in our study differed compared to ABX treatment 3–5 days pre-partum to euthanasia of pups (Gonzalez-Perez et al., 2016). Of interest, immune cell defects were partially restored by oral LPS administration during VV infection of pups [30]. In our study, we speculate that cessation of maternal ABX treatment on the day pups were infected with IAV may have allowed for re-colonization of pups to a sufficient degree to ensure responsiveness to IAV infection, similar to our observations in juvenile ABX2-treated mice.

In conclusion, our study demonstrates that ABX treatment of juvenile mice changes their GM composition and this was associated with a delayed gain in body weight only during the time of treatment. In contrast, perinatal ABX treatment was shown to have long-term consequences for post-natal growth of pups. Furthermore, we demonstrate that ABX treatment of juvenile mice prior to, and during, IAV infection results in enhanced susceptibility and reduced acute CD8^+^ T cell responses. However, when ABX treatment is ceased at the time of IAV infection, this is sufficient to allow for rapid GM re-colonization and restoration of immunity to IAV infection. Finally, perinatal ABX treatment did not result in any overt effects on pups in regard to susceptibility and immunity to IAV infection.

## Data availability statement

All datasets generated for this study are included in the manuscript and the supplementary files.

## Author contributions

EF, AP, AB, HF, and PR designed research. EF, AP, LK, and PR performed research. EF, AP, and LK analyzed data. EF, AP, LK, DN, HF, and PR wrote the paper.

### Conflict of interest statement

The authors declare that the research was conducted in the absence of any commercial or financial relationships that could be construed as a potential conflict of interest.
