# Supplementary material for: Changes in Gut Microbiota Prior to Influenza A Virus Infection Do Not Affect Immune Responses in Pups or Juvenile Mice
Source: Front Cell Infect Microbiol. 2018 Sep 12;8:319. doi: 10.3389/fcimb.2018.00319 (PMC6145060; doi:10.3389/fcimb.2018.00319)
Supplement: Supplementary file 1 [file Data_Sheet_1.docx]

**Supporting information**

**
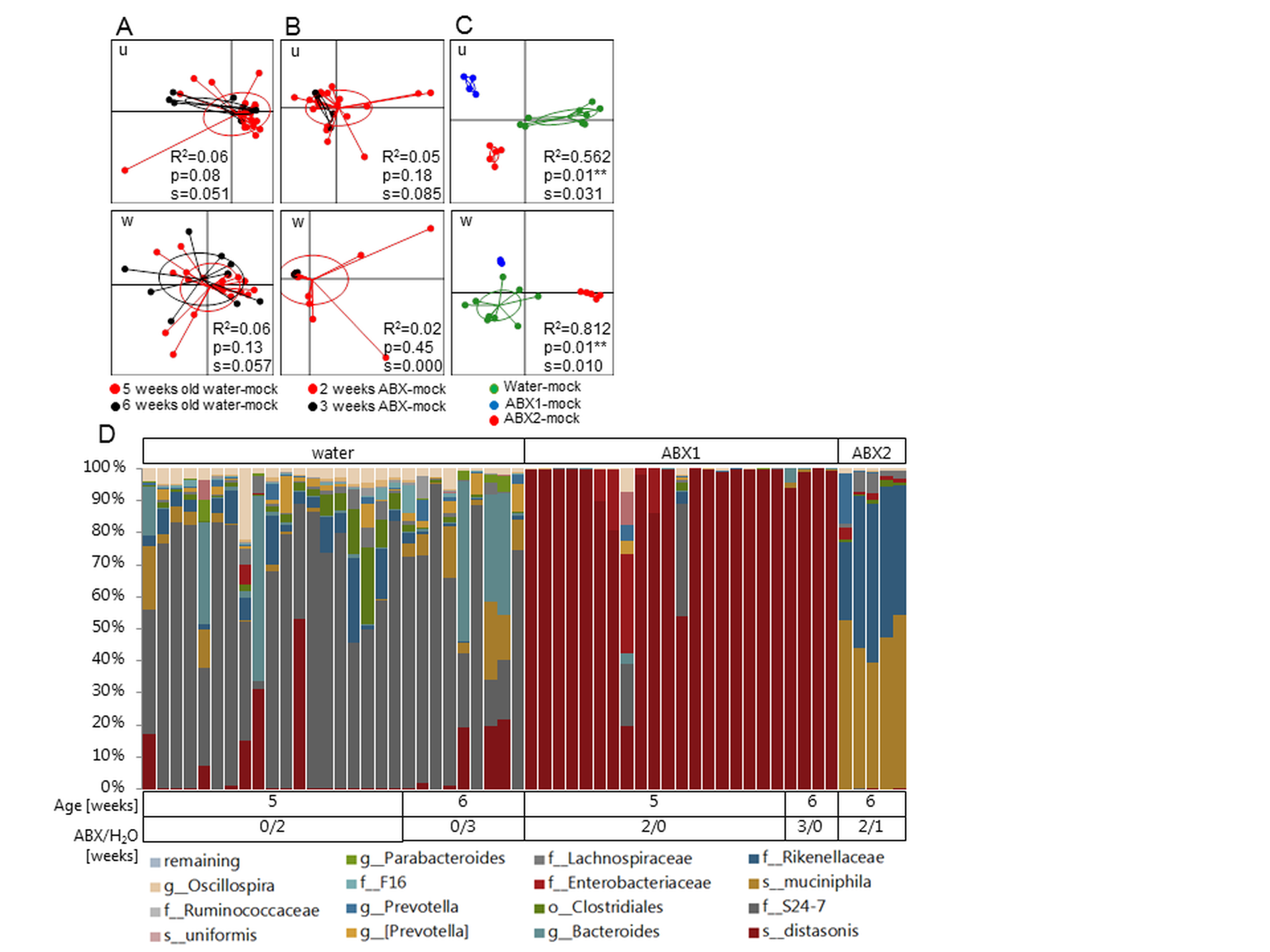
**

**S1 Fig. GM in juvenile mice.** NMDS plots based on unweighted (u) and weighted (w) UniFrac distance matrices showing similarities in GM composition of juvenile (A) water-treated mice at 5 weeks of age (red, n = 19) compared to at 6 weeks of age (black, n = 9), and (B) mice treated with ABX for 2 weeks (red, n = 19) compared to 3 weeks (black, n = 4). (C) GM from the three juvenile groups: water (green, n = 9), ABX1 treatment (blue, n = 4), 2 weeks of treatment followed by one week back on water (ABX2, red, n = 5). PERMANOVA results are presented in bottom right corners (R^2^ and p values). S values represent the stress of each NMDS ordination. (D) Bar-chart depicting relative abundances of the fifteen most dominating bacterial taxa (remaining taxa were summarized within one category).

**
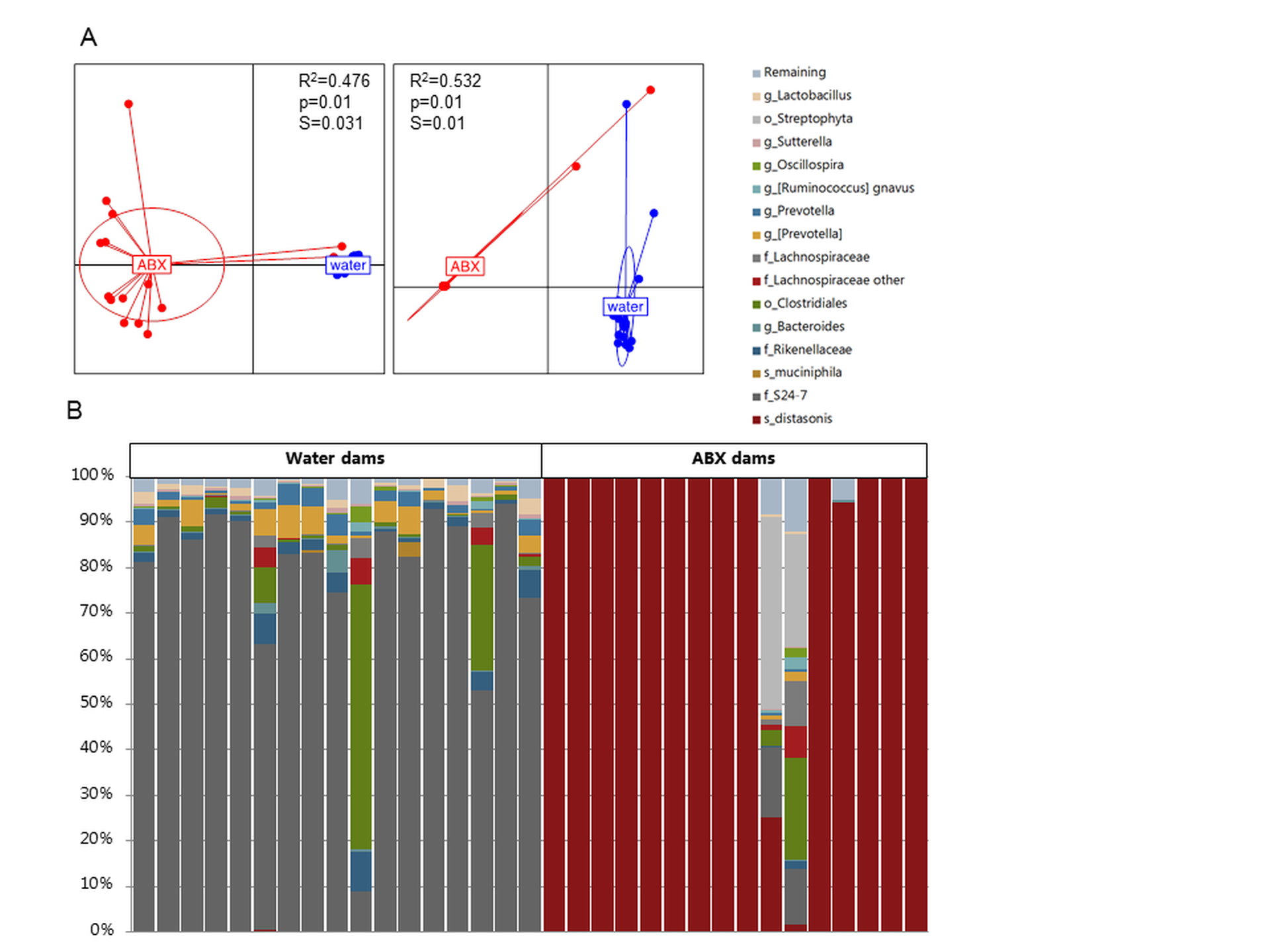
**

**S2 Fig. GM in dams and their pups.** (A) GM composition was compared with NMDS plots between water-treated dams (green, n = 18) and ABX-treated dams (blue, n = 18). PERMANOVA results are presented inside the plots (R^2^ and p values). S values represent the stress of each NMDS ordination. (B) Bar-chart depicting relative abundances of the fifteen most dominating bacterial taxa (remaining taxa were summarized within one category).


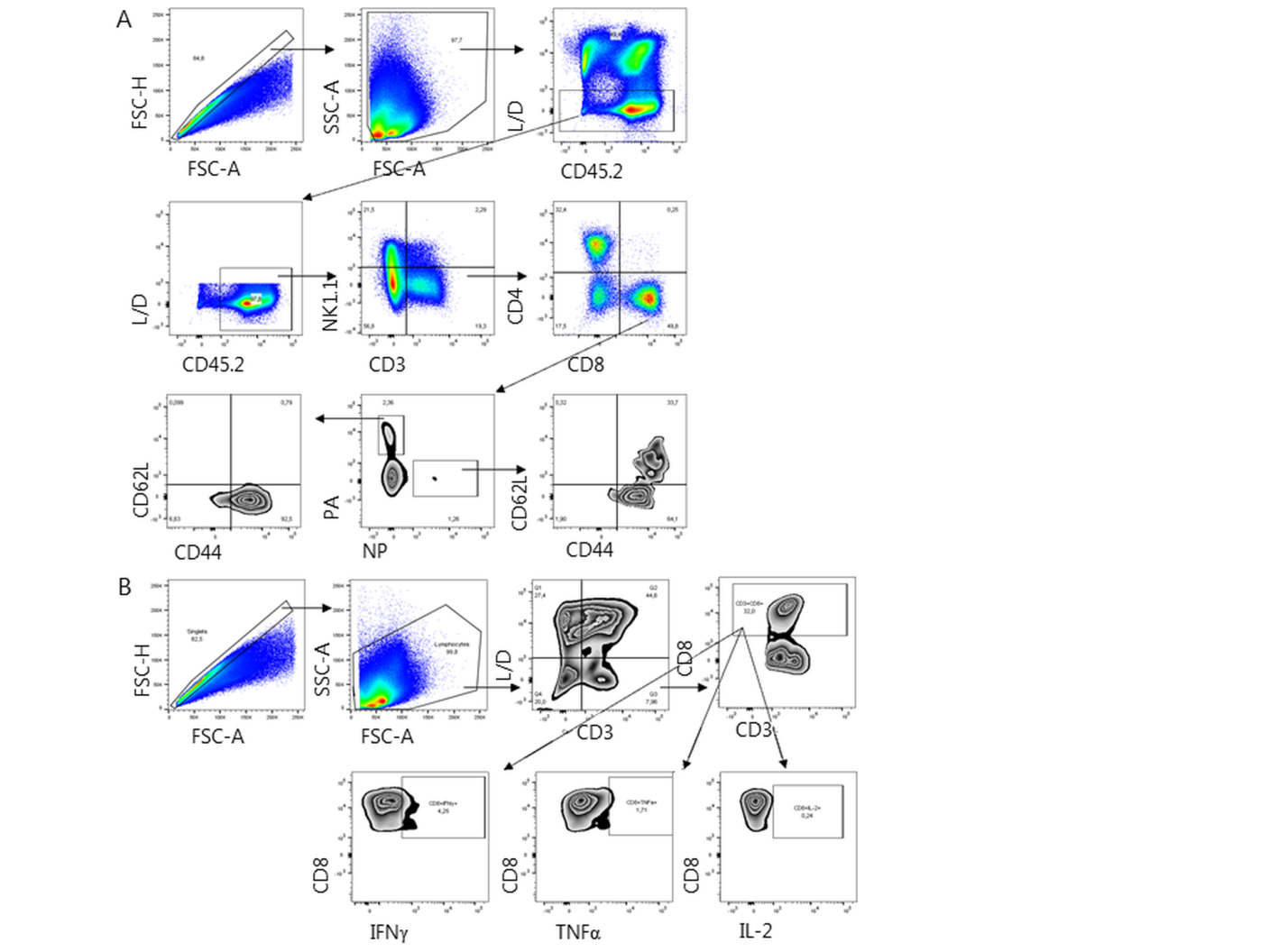


**S3 Fig.** **Gating strategy for surface staining and intracellular cytokine staining.** (A) Single cell suspensions prepared from lung and spleen were analyzed by flow cytometry using the following gating strategy: Single cells (FSC-A/FSC-H) 🡪 lymphocytes (FSC-A/SSC-A) 🡪 Living cells (L/D^-^CD45.2) 🡪 CD45.2^+^ (L/D^-^CD45.2^+^) 🡪 NK cells (NK1.1^+^CD3^-^), CD3 cells (NK1.1^-^CD3^+^) 🡪 CD3^+^CD4^+^ T cells (CD4^+^CD8^-^), CD3^+^CD8^+^ T cells (CD4^-^CD8^+^) 🡪 CD8^+^NP^+^ (PA^-^NP^+^), CD8^+^PA^+^ (PA^+^NP^-^) 🡪 CD8^+^NP^+^/PA^+^ T/T_EM_ (CD62L^-^CD44^+^), CD8^+^NP^+^/PA^+^ T_CM_ (CD62L^+^CD44^+^). (B) Intracellular cytokine production in lung and spleen cell suspensions was analyzed by flow cytometry using the following gating strategy: Single cells (FSC-A/FSC-H) 🡪 lymphocytes (FSC-A/SSC-A) 🡪 Living T cells (L/D^-^CD3^+^) 🡪 CD3^+^CD8^+^ T cells (CD3^+^CD8^+^) 🡪 CD8^+^IFNγ^+^ (CD8^+^IFNγ^+^), CD8^+^TNFα^+^ (CD8^+^TNFα^+^ ), CD8^+^IL-2^+^ (CD8^+^IL-2^+^).


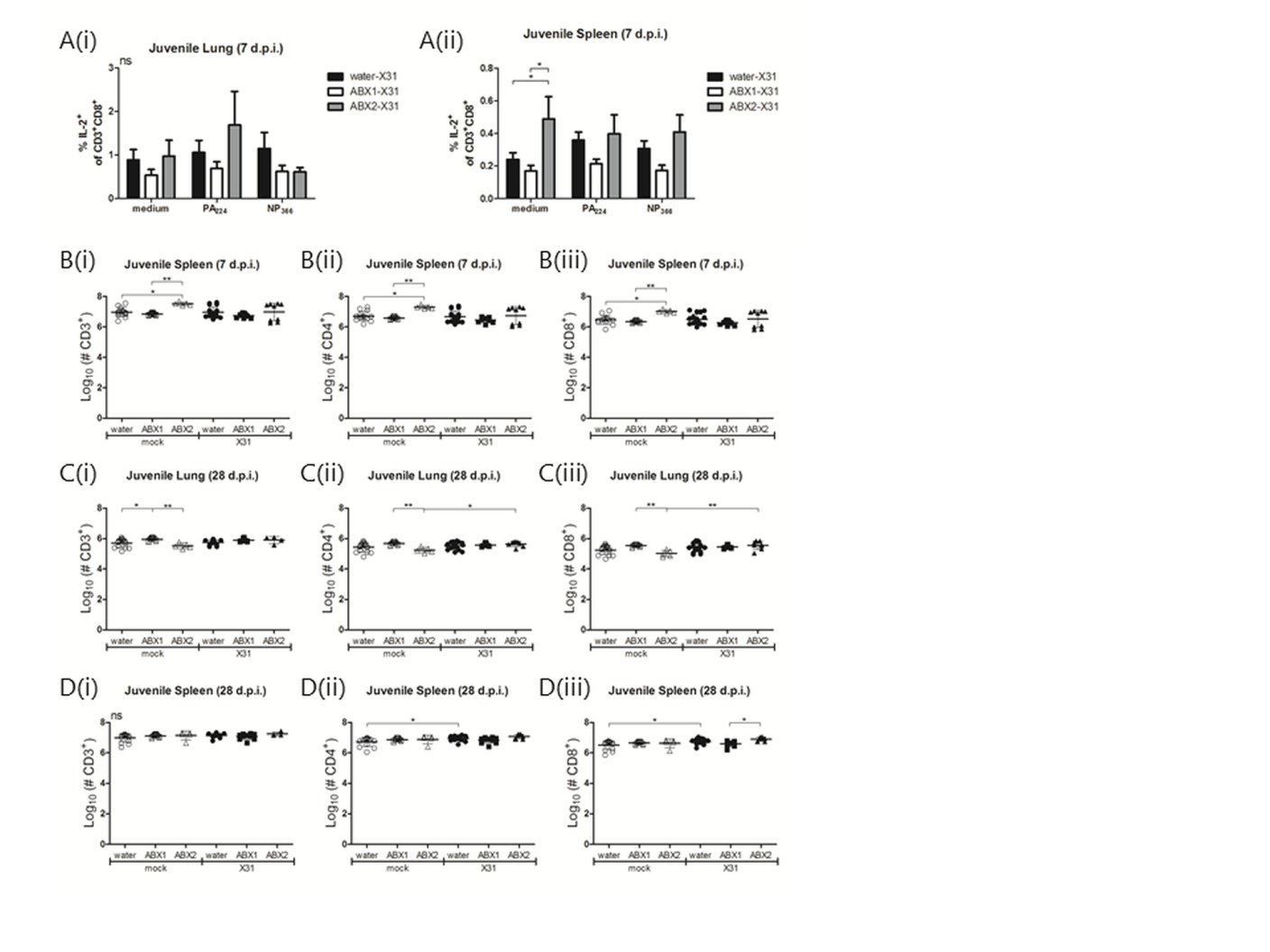


**S4 Fig. Effect of antibiotic treatment prior to and during, or only prior to infection, on lung and spleen lymphocyte responses in juvenile mice.** At day 7 and 28 p.i., single cell suspensions prepared from the lungs and spleen were analyzed by flow cytometry. (A) Intracellular cytokine staining was performed following incubation of (i) lung and (ii) spleen cell suspensions with PA/NP-specific peptides, or with medium alone. The percentage of CD8^+^ T lymphocytes recovered at day 7 p.i. and staining positive for intracellular IL-2 are shown from water-X31 (black bars, n=15), ABX1-X31 (white bars, n=9) and ABX2-X31 (grey bars, n=5) mice. (B-D) Numbers of (i) total CD3^+^, (ii) CD3^+^CD4^+^ and (iii) CD3^+^CD8^+^ lymphocytes: (B) day 7 p.i. spleen, (C) day 28 p.i. lung, (D) day 28 p.i. spleen. (A) Data show the mean ± SD. (B-D) results from individual animals are shown with the horizontal lines depicting the mean ± SD. For day 7 p.i., data represent results from water-mock (n=15), ABX1-mock (n=10), ABX2-mock (n=5), water-X31 (n=20), ABX1-X31 (n=10) and ABX2-X31 (n=10). For day 28 p.i., data represent results from water-mock (n=15), ABX1-mock (n=10), ABX2-mock (n=5), water-X31 (n=20), ABX1-X31 (n=10) and ABX2-X31 (n=10). For statistical analyses, data were analyzed using either one-way ANOVA (B-D) or two-way ANOVA (A), followed by Bonferroni’s post-test. ****p*<0.001, ***p*<0.01, **p*<0.05, ns = not significant.


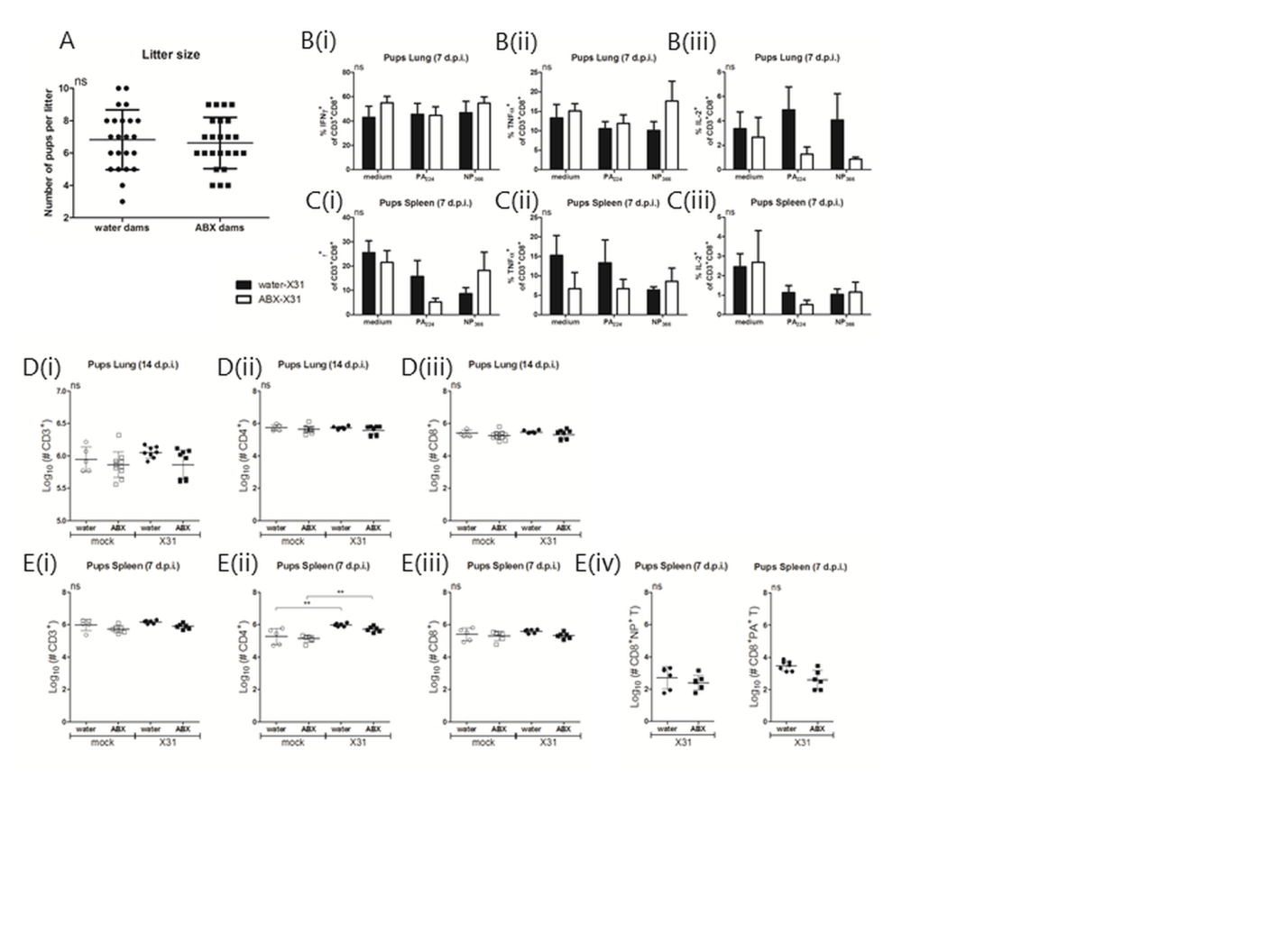


**S5 Fig. Effect of antibiotic treatment of pregnant dams on litter size and acute CD8^+^ T cell responses in pups following X31 infection.** (A) On the day of birth, the number of pups per litter from ABX or water dams were determined. Results from individual litters are shown with horizontal lines depicting the mean ± SD. Data represent results from water dams (n=23) and ABX dams (n=24). At day 7 and 14 p.i., single cell suspensions prepared from (B/D) lungs and (C/E) spleen were analyzed by flow cytometry. Intracellular cytokine staining was performed following incubation of (B) lung and (C) spleen cell suspensions with PA/NP-specific peptides, or with medium alone. The percentage of day 7 p.i. CD8^+^ T lymphocytes staining positive for intracellular (i) IFNγ, (ii) TNFα, or (iii) IL-2 are shown from water-X31 (black bars, n=11) and ABX-X31 (white bars, n=9). Data are shown as the mean ± SD and were analyzed using two-way ANOVA. Number of (D) day 14 p.i. lung lymphocytes, and numbers of day 7 p.i. (E(i-iii)) spleen lymphocytes and (E(iv)) NP- and PA-specific CD8^+^ T cells recovered from the spleen after infection. For (D-E), results from individual animals are shown with the horizontal lines depicting the mean ± SD. Data represent results from day 7 or 14 for water-mock (n=5, 10, respectively), ABX-mock (n=6, 12, respectively), water-X31 (n=11, 8, respectively) and ABX-X31 (n=9, 9, respectively). Data were analyzed using one-way ANOVA followed by Bonferroni’s post-test (i-iii) or Student’s t-test (iv). ****p*<0.001, ***p*< 0.001, **p*<0.05, ns = not significant.
